# A genetic regulatory see-saw of biofilm and virulence in MRSA pathogenesis

**DOI:** 10.3389/fmicb.2023.1204428

**Published:** 2023-06-22

**Authors:** Hardi Patel, Seema Rawat

**Affiliations:** Microbiology Laboratory, School of Life Sciences, Central University of Gujarat, Gandhinagar, Gujarat, India

**Keywords:** methicillin-resistant *Staphylococcus aureus* (MRSA), pathogenesis, biofilm, virulence, two-component system (TCS)

## Abstract

*Staphylococcus aureus* is one of the most common opportunistic human pathogens causing several infectious diseases. Ever since the emergence of the first methicillin-resistant *Staphylococcus aureus* (MRSA) strain decades back, the organism has been a major cause of hospital-acquired infections (HA-MRSA). The spread of this pathogen across the community led to the emergence of a more virulent subtype of the strain, i.e., Community acquired Methicillin resistant *Staphylococcus aureus* (CA-MRSA). Hence, WHO has declared *Staphylococcus aureus* as a high-priority pathogen. MRSA pathogenesis is remarkable because of the ability of this “superbug” to form robust biofilm both *in vivo* and *in vitro* by the formation of polysaccharide intercellular adhesin (PIA), extracellular DNA (eDNA), wall teichoic acids (WTAs), and capsule (CP), which are major components that impart stability to a biofilm. On the other hand, secretion of a diverse array of virulence factors such as hemolysins, leukotoxins, enterotoxins, and Protein A regulated by *agr* and *sae* two-component systems (TCS) aids in combating host immune response. The up- and downregulation of adhesion genes involved in biofilm formation and genes responsible for synthesizing virulence factors during different stages of infection act as a genetic regulatory see-saw in the pathogenesis of MRSA. This review provides insight into the evolution and pathogenesis of MRSA infections with a focus on genetic regulation of biofilm formation and virulence factors secretion.

## 1. Introduction

*Staphylococcus aureus* is a normal microflora of the human nasal cavity and skin but may cause diseases such as skin and soft tissue infections, endocarditis, osteomyelitis, bacteremia, and lethal pneumonia on disruption of mucosal and cutaneous barriers due to surgical procedures, wounds, or chronic skin conditions. It is considered as one of the most common opportunistic human pathogens among immunocompromised patients, children, elderly, and patients with medical devices. Penicillin was extensively prescribed by doctors to cure *S. aureus* infections until Penicillin-resistant *S. aureus* was reported in the 1950's. To overcome this, in 1959 scientists developed a semisynthetic penicillin named methicillin which was resistant to penicillinase (Guo et al., 2020). Only after 2 years of the introduction of methicillin for clinical use, i.e., in 1961, a British scientist named Jevons isolated the first methicillin-resistant *Staphylococcus aureus* (MRSA) strain in Europe. MRSA possessed a gene *mecA* which was responsible for encoding the penicillin-binding protein 2a or 2′ (PBP2a or PBP2′). This gene was integrated into the chromosomal element (SCCmec) of methicillin-sensitive *Staphylococcus aureus* (MSSA). Since then, MRSA has been identified as one of the most notorious human pathogens across the world (Guo et al., 2020).

It is still debatable whether MRSA is more virulent than MSSA. Meta-analysis results of some of the epidemiological studies have indicated increased mortality and/or morbidity in the case of nosocomial MRSA infections such as those associated with surgery, pneumonia, and bloodstream, etc. as compared to MSSA infections (Cosgrove et al., 2003; Engemann et al., 2003; Gastmeier et al., 2005; Reed et al., 2005). Increased mortality by MRSA bacteremia as compared to MRSA pneumonia has been reported by Shurland et al. (2007). However, other studies reported no significant difference in mortality associated with nosocomial MRSA bacteremia (Cosgrove et al., 2005) or ventilator-associated pneumoniae (Zahar et al., 2005) as compared to those caused by MSSA. Another study comparing CA-MSSA and CA-MRSA skin infections also did not report any serious outcomes caused by the latter (Miller et al., 2007). To date, no study provides clear evidence that MRSA is more virulent than MSSA; however, treatment of invasive MRSA infections is challenging due to the lack of antibiotics and increased treatment costs (Gordon and Lowy, 2008).

Initially, MRSA was only associated with hospital outbreaks, more commonly termed as hospital-acquired methicillin-resistant *Staphylococcus aureus* (HA-MRSA). It was found to be responsible for 20–80% of secondary hospital infections (Krishnamurthy et al., 2014; Kemung et al., 2018). *agr* system, responsible for producing toxins for exhibiting virulence, is generally found to be either less active or mutated in HA-MRSA strains leading to high-level expression of adhesins, which aids in robust biofilm formation on implanted medical devices and are also more resistant to antibiotics (Painter et al., 2014; Suzuki et al., 2015).

Approximately 20 years after the first reported case of MRSA, community-acquired methicillin-resistant *Staphylococcus aureus* (CA-MRSA) was reported in Detroit, Michigan, USA in 1980 (Saravolatz et al., 1982). According to the Center for Disease Control and Prevention (CDC), CA-MRSA can be defined as an infection from an MRSA culture isolated within 48 h of admission of a patient in a hospital with no previous history of hospital admission or medical treatment with surgical procedures (Gorwitz et al., 2006). According to a CDC report of 2016, two out of 100 people are carriers of CA-MRSA, suggesting its ability to spread more easily than HA-MRSA. The presence of more mobile genetic elements *viz*., novel SCCmec elements, Panton-Valentine Leukocidin (PVL)-encoding genes, and ACME (arginine catabolite mobile element) in CA-MRSA is considered to be the plausible cause of more virulence observed in it as compared to HA-MRSA (Udo and Boswihi, 2017; Boswihi and Udo, 2018).

Recently, the evidence of the spread of MRSA from animals to humans led to the conclusion that animals are reservoirs as well as potent carriers of MRSA, which is a serious threat to human health (Voss et al., 2005; Loncaric et al., 2013; Van Boeckel et al., 2015). The plausible reason for the emergence of live-stock-associated methicillin-resistant *Staphylococcus aureus* (LA-MRSA) is the overuse of antibiotics for increasing the yield in live-stock production (Van Boeckel et al., 2015).

## 2. Pathogenesis of MRSA

**Figure 1:**
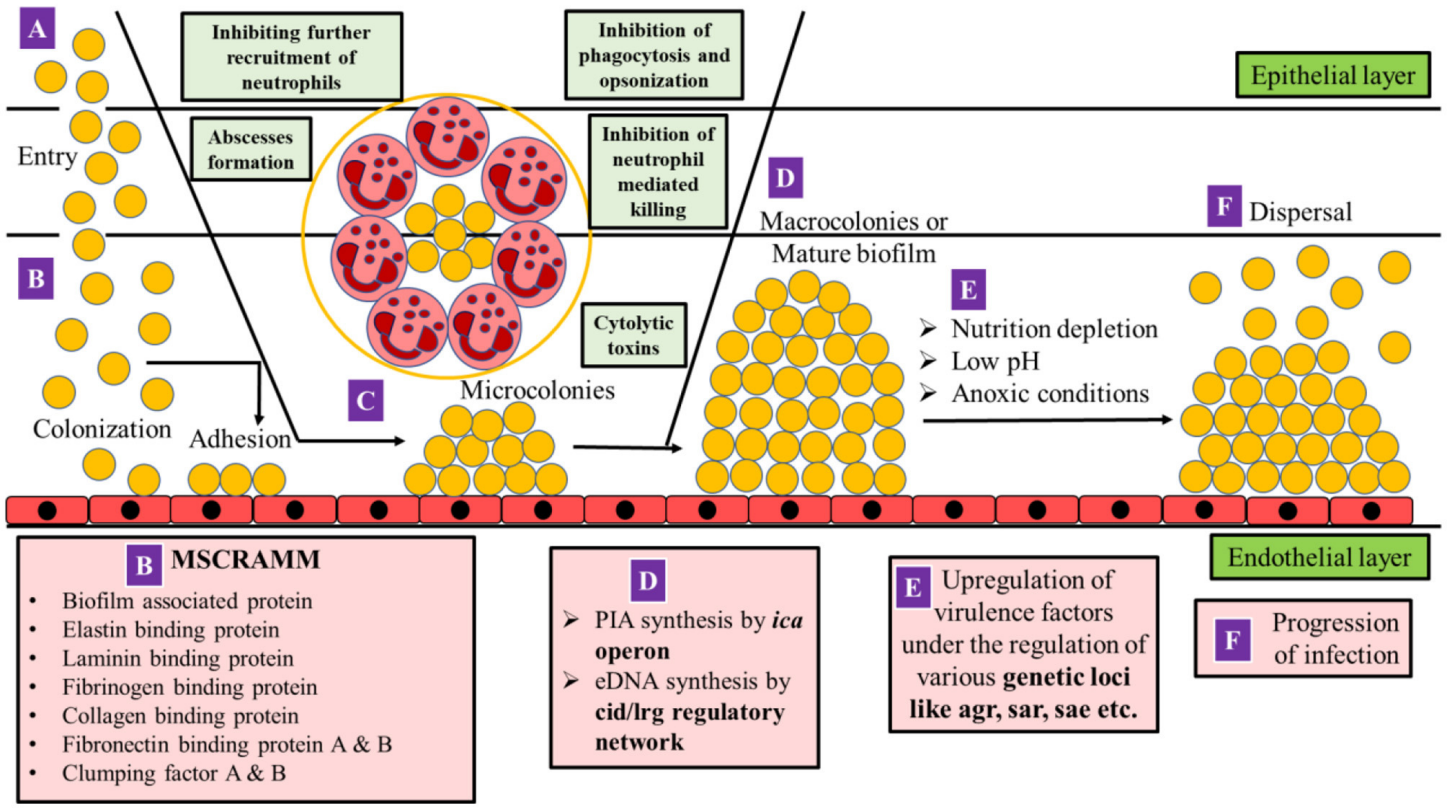
Mechanism of pathogenesis in MRSA. **(A)** *S. aureus* gains access to the bloodstream or underlying tissues due to a breach in cutaneous or mucosal barriers. **(B)** Colonization occurs on the endothelial layer by adhesion with the help of MSCRAMM. **(C)** As the *S. aureus* cells multiply, polymorphonuclear leukocytes (PMNs) are recruited, and a fibrin pseudo-capsule termed as abscess is formed with the help of coagulase proteins secreted by *S. aureus* which surrounds bacterial cells and recruited (PMNs). *S. aureus* can inhibit the further entry of PMNs at the site of infection, opsonization, phagocytosis, and neutrophil-mediated killing. The bacterium which is already phagocytosed by PMNs releases cytolytic toxins such as hemolysins, leukocidins such as Panton-Valentine Leukocidin (PVL) and PSM peptides to escape the host defense. The bacterial cells further multiply leading to the formation of microcolonies on the endothelial layer. **(D)** Synthesis of poly intracellular adhesin (PIA) and extracellular DNA (eDNA) is initiated by the *ica* operon and *cid/lrg* regulatory network, respectively, resulting in the formation of macrocolonies or a mature biofilm. **(E)** As biofilm matures, the core becomes anoxic, nutrient-deprived, and acidic in pH which triggers the upregulation of various virulence factors (*agr, sar*, and *sae*), which mediates **(F)** dispersal of biofilm as well as the progression of infection (adapted from Moormeier and Bayles, 2017; Sedarat and Taylor-Robinson, 2022).

The entry of *S. aureus* into the endothelial tissues of the host begins when the bacterium, from an external source or from indigenous microflora, gains access to the bloodstream or underlying tissues leading to infections (Figure 1).

### 2.1. Mechanism of pathogenesis

#### 2.1.1. Colonization

The colonization of the mammalian cell's extracellular matrix proteins (fibrinogen, fibronectin, elastin, collagen, laminin, and vitronectin) occurs covalently or non-covalently, mediated by several molecules of *S. aureus* that are collectively termed as microbial surface components recognizing adhesive matrix molecules (MSCRAMMs; Table 1). Teichoic acids (TAs), which are common components of Gram-positive bacterial cell wall, also contribute significantly to the adherence to host cells (Qin et al., 2007). Approximately 15% of individuals (persistent carriers) have been reported to have continuous *S. aureus* colonization, whereas 70% of individuals have intermittent colonization (frequent *S. aureus* infection but immediate eradication), and the remaining 15% of individuals are non-carriers (Eriksen et al., 1995).

**Table 1:** Microbial surface components recognizing adhesive matrix molecules (MSCRAMMs).

**Adhesion protein**	**Encoding gene**	**Function**	**References**
Biofilm-associated protein	*bap*	Promotes the formation of biofilm	Cucarella et al., 2001
Elastin-binding protein	*ebpS*	Promotes the binding of soluble elastin peptides and tropo- elastin to *S. aureus* cells, however, it is not able to promote bacterial adherence to immobilized elastin and, therefore, is not a microbial surface component recognizing adhesive matrix molecule (MSCRAMM)	Park et al., 1991; Roche et al., 2004
Laminin-binding protein	*eno*	Binds to laminin by destructing extracellular matrix on the host cell leading to invasion and dissemination	Carneiro et al., 2004
Fibrinogen-binding protein	*fib*	Interacts with alpha chain of fibrinogen and its derivative, fibrin, and causes repression of fibrinogen-dependent platelet aggregation	Palma et al., 2001
Collagen-binding protein	*cna*	Facilitates bacterial adherence to collagenous tissues such as cartilage of host cell	Patti et al., 1993
Fibronectin-binding protein A	*fnbA*	Promotes bacterial attachment to both soluble and immobilized forms of fibrinogen (Fg) by means of a unique binding site localized within the 17 C-terminal residues of the gamma-chain of human Fg. Both plasma proteins (Fn and Fg) function as a bridge between the bacterium and host cell	Wann et al., 2000
Fibronectin-binding protein B	*fnbB*	Multifunctional protein which promotes bacterial attachment to fibrinogen, elastin, and fibronectin	Roche et al., 2004; Burke et al., 2011; Pietrocola et al., 2016
Clumping factor A	*clfA*	Promotes bacterial attachment to the gamma-chain of human fibrinogen making it a dominant factor responsible for human platelet aggregation	Siboo et al., 2001
Clumping factor B	*clfB*	Promotes bacterial attachment to both alpha- and beta-chains of human fibrinogen, mediates bacterial attachment to the highly keratinized squamous epithelial cells from the nasal cavity *via* interaction with cytokeratin K10 (K10)	Ní Eidhin et al., 1998; O'Brien et al., 2002

Apart from the host- and pathogen-associated factors, various other factors also play roles in *S. aureus* colonization. The nasal cavity is one of the most important sites of colonization for *S. aureus* as nose-picking can lead to the spread of bacteria to other body parts as well as to other hosts (Von Eiff et al., 2001; Wertheim et al., 2004, 2006). The nasal microbiota (*Corynebacterium* spp., *Propionibacterium acnes, Staphylococcus epidermis*, and *Staphylococcus lugdunensis*) and the invading pathogen *S. aureus* compete among themselves in various ways for colonization. Nutrition is one of the major limiting factors for colonization in the human nose and therefore *S. aureus* has been found to better adapt than coagulase-negative staphylococci due to the ability of the former to thrive in the low-nutrient environment (Lemon et al., 2010; Krismer et al., 2014; Liu et al., 2015; Lee et al., 2018).

The growth of *S. aureus* including MRSA has been found to be inhibited by the indigenous nasal bacterium *Staphylococcus lugdunensis* both *in vitro* and *in vivo* due to the production of an antimicrobial compound called lugdunin that can rapidly breakdown bacterial energy resources (Zipperer et al., 2016). The risk of colonization of *S. aureus* has been reported to be 6-fold lower in humans with *Staphylococcus lugdunensis* in their nasal cavities. However, nasal colonization with *Staphylococcus lugdunensis* has been reported in only 9–26% of the general population (Kaspar et al., 2016; Zipperer et al., 2016; Lee et al., 2018). *S. aureus* has been reported to compete with other commensal bacteria by the activation of host defense *via* upregulation of the production of antimicrobial proteins that are less harmful to it than to others (Krismer et al., 2017; Lee et al., 2018). Apart from the nasal cavity, other sites in the human body where *S. aureus* colonizes are armpits (8%), abdomen (15%), intestine (17–31%), perineum (22%), vagina (5%), and pharynx (4–64%; González-García et al., 2023). The virulence genes responsible for toxin production in *S. aureus* are downregulated during the colonization process.

The multiplication of bacteria after colonization on both biotic and abiotic surfaces leads to the formation of a three-dimensional complex community of bacteria within a layer of exopolysaccharide (EPS) termed as “biofilm” (Figure 1; Costerton et al., 1999; Sedarat and Taylor-Robinson, 2022). The accumulation of biofilm is facilitated by the formation of microcolonies due to the production of exopolysaccharide (a major component of biofilm), which comprises 97% water and 3% of structural and functional molecules (Guzmán-Soto et al., 2021). EPS of biofilm contains both positively and negatively charged groups as well as hydrophobic groups. The negatively charged groups of EPS include phosphates, sulfates, carboxyl groups, glutamic acid, and aspartic acid, whereas the positively charged groups include amino sugars such as polysaccharide intercellular adhesin (PIA). Despite positively charged PIA being a major component, the overall charge on the EPS surface is negative, serving as a better target site for positively charged drug candidates (Idrees et al., 2021).

The major EPS produced by *S. aureus* is polysaccharide intercellular adhesin (PIA), also known as poly-*N*-acetyl-glucosamine (PNAG; Mack et al., 1996). PIA/PNAG has a net positive charge and it promotes intercellular interactions by binding to the negatively charged surfaces of bacterial cells such as teichoic acids (O'Gara, 2007; Vergara-Irigaray et al., 2008). The multivalent electrostatic interaction of the cationic PIA polymer with the negatively charged wall teichoic acids on staphylococcal cells as revealed by single-cell force spectroscopy confirmed that the cationic nature of PIA is crucial for its attachment to the cell surface and intercellular adhesion (Formosa-Dague et al., 2016). PIA mutants exhibited a decreased ability to adhere to each other (Peng et al., 2022). There is no evidence for a covalent linkage of PIA to the cell surface (Cue et al., 2012). PIA is evident for biofilm formation under high-shear flow conditions such as those found inside catheters as compared to low-shear conditions such as those in subcutaneously implanted tissue, ocular infections, or platelet concentrate (Nguyen et al., 2020).

In addition to PIA/PNAG, biofilms contain bacterial proteins, eDNA, ions, and carbohydrates (Guzmán-Soto et al., 2021) as essential components with their ratios being variable. A number of staphylococcal strains exhibit PIA/PNAG-independent biofilm formation where the secreted proteins and extracellular DNA substitute for PIA/PNAG (Cue et al., 2012).

Extracellular DNA (eDNA) is another important structural component of biofilm matrix serving as a “glue” for the community due to its adhesive property. The destabilization of a matured biofilm by using DNAase I in *Pseudomonas aerugionosa* reported by Whitchurch et al. (2002) suggested the role of eDNA in biofilm formation in pathogenic bacteria. In a matured biofilm when there is a scarcity of nutrients, a subsequent population of damaged cells is eliminated in order to release nutrients for healthy cells. This self-destructive or suicidal act of cells is termed as autolysis or programmed cell death (PCD; Lewis, 2000). Along with the nutrients, the lysed cells also release genomic DNA in the form of eDNA. Rice et al. (2007) reported that only a small fraction of the bacterial population (< 1%) within the biofilm undergoes cell lysis to release a sufficient amount of eDNA required for biofilm stability (Bayles, 2007). eDNA can also confer antibiotic resistance mainly due to the horizontal gene transfer of eDNA to the healthy cells of biofilm (Molin and Tolker-Nielsen, 2003; Tetz et al., 2009).

Capsular polysaccharides (CPs) are long-chain polysaccharides attached covalently to the peptidoglycan layer of the cell wall. They aid in the colonization of the host as well as in biofilm formation, hence are indirectly involved in the progression of invasive diseases. However, the contrasting effect on the virulence of *S. aureus* is observed by the presence or absence of a capsule depending upon the type of infection (O'Riordan and Lee, 2004; Tuchscherr et al., 2010). CP enhances virulence in murine models of bacteremia (Thakker et al., 1998; Watts et al., 2005), septic arthritis (Nilsson et al., 1997), abscess formation (Portols et al., 2001), and surgical wound infection (McLoughlin et al., 2006). On the contrary, in mammary gland infections (Tuchscherr et al., 2005) and in catheter-induced endocarditis (Baddour et al., 1992; Nemeth and Lee, 1995), CP mutants are more virulent. This is likely because CP also inhibits the adherence of the underlying adhesins to their specific target molecule (Phlmann-Dietze et al., 2000; Risley et al., 2007). CP-negative *S. aureus* strains are frequently isolated from patients with osteomyelitis, mastitis, or cystic fibrosis, providing evidence that the loss of CP expression (due to mutations in any of the genes essential for CP biosynthesis or in the promoter region; Cocchiaro et al., 2006; Tuchscherr et al., 2010) may be advantageous for *S. aureus* during chronic infection (Herbert et al., 1997; Lattar et al., 2009; Tuchscherr et al., 2010). CPs also help bacteria in evading the phagocytic uptake by the host immune system and also protect the important bacterial cell wall constituents (Berni et al., 2020). Out of 13 serotypes of *S. aureus* (Berni et al., 2020), serotypes 1 and 2 (rarely reported among clinical isolates) produce mucoid colonies while the remaining serotypes produce non-mucoid colonies on a solid medium. Serotypes 5 and 8 (prevalent among clinical isolates as well as commensal sources) constitute ~25 and 50%, respectively, of the isolates recovered from humans from various geographic locations of the world (O'Riordan and Lee, 2004).

Teichoic acids are one of the major components of Gram-positive bacterial cell wall. These diverse anionic carbohydrate-based polymers are categorized into two classes: (1) lipoteichoic acids (LTAs), which are embedded in the lipid bilayer with the help of a diacylglycerol lipid anchor that can extend from the cell surface to the peptidoglycan layer, and (2) wall teichoic acids (WTAs), which are covalently attached to peptidoglycan matrix *via* a phosphodiester linkage to the C6 hydroxyl of the N-acetyl muramic acid sugars and can extend through and beyond the cell wall (Swoboda et al., 2010; Berni et al., 2020). WTAs comprise ~60% of the total cell mass of Gram-positive bacteria. *S. aureus* WTAs are essential for adhesion to host tissues as well as to artificial surfaces including glass and polystyrene (Gross et al., 2001). WTAs null mutants are defective in their ability to produce biofilm; however, the reduced production of PNAG (an inevitable component of biofilm) was not reported, suggesting an independent role of WTAs in biofilm formation (Vergara-Irigaray et al., 2008). WTA mutant *S. aureus* strains were also unable to adhere and colonize the nasal epithelial cells as well as endothelial tissues of the kidney and the spleen derived from cotton rats. D-alanylation, which takes place on LTA, was reported to be intact on these strains, implying that WTAs were independently involved in cell adhesion and eventually biofilm formation (Weidenmaier et al., 2005). The involvement of WTAs in host colonization, infection, and biofilm formation makes it an important virulence factor and the enzymes involved in its biosynthesis can be novel targets for the discovery of new antimicrobials (Weidenmaier and Peschel, 2008).

Signaling molecules such as cyclic AMP (cAMP) are not only linked with the regulation of carbon metabolism and stringent response but also with the expression of virulence genes and biofilm formation in Gram-positive bacteria (Schilcher and Horswill, 2020). *S. aureus* requires two molecules of ATP and the enzyme diadenylyl cyclase DacA to synthesize c-di-AMP. Phosphodiesterase GdpP is involved in the degradation of c-di-AMP (Corrigan et al., 2011). Screening for essential genes of *S. aureus* revealed that *dacA* disruption was not possible, indicating the importance of c-di-AMP formation for the viability of the bacterium (Chaudhuri et al., 2009). On the other hand, *gdpP* deletion in the *S. aureus* SEJ1 strain yielded a 3-fold increase in biofilm formation, highlighting the fact that high levels of c-di-AMP induced biofilm formation in this strain. However, similar experimental conditions did not replicate the results in *S. aureus* USA300 LAC or its corresponding *gdpP* mutant (Corrigan et al., 2011). Single nucleotide polymorphisms (SNPs) or deletions in *gdpP* in various homogenous oxacillin-resistant (HoR) *S. aureus* isolates exhibited a decrease in the expression of *icaADBC* and *agr* and an increase in the expression of penicillin-binding protein 2 (PBP2). This resulted in an absence of polysaccharide content in the biofilm and the formation of a more proteinaceous biofilm (Pozzi et al., 2012). Apart from this, *gdpP* mutants were also impaired in their ability to form eDNA suggesting that c-di-AMP is essential for eDNA release as well (DeFrancesco et al., 2017). The role of c-di-AMP in biofilm formation may be strain dependent as different studies yielded different biofilm phenotypes in *gdpP* mutants of *S. aureus* (Schilcher and Horswill, 2020).

*S. aureus* possesses small α-helical peptides called phenol soluble modulins (PSM) which act as surfactants and disrupt cell–cell interactions within the biofilm better than proteases, unlike other bacteria that commonly use nucleases and proteases for biofilm detachment (Otto, 2008). The detachment and dispersal of planktonic cells are mediated by changes conferred in pH, nutrition, waste accumulation, oxygen depletion, etc. (Otto, 2008).

#### 2.1.2. Invasion and infection

Once colonization is established, the bacteria adhere and start multiplying on wounded tissues, resulting in the upregulation of virulence genes and the production of toxins that further aid in disease progression. The initially expressed adhesion genes are now downregulated (Novick, 2003; Foster et al., 2014).

During the onset of infection, 95% of iron is within host cells in the form of serum iron bound to host proteins. In such a situation of iron starvation, *S. aureus* secretes high-affinity iron-binding proteins such as staphyloferrin (Drechsel et al., 1993) and aureochelin (Courcol et al., 1997). *S. aureus* can also initiate transcription of Isd (iron surface determinants)-mediated heme-iron transport that facilitates the release of heme from hemoglobin, haptoglobin, and hemopexin. This free heme transports across the plasma membrane *via* the iron complex and free iron is released in the cytoplasm of bacterial cell as a result of oxidative degradation (Maresso and Schneewind, 2006). This helps the bacterium in survival along with evasion from host defense (Liu, 2009).

Gram-positive bacteria including *S. aureus* secrete a diverse array of chemo-attractants such as peptidoglycan (Dziarski and Gupta, 2005), N-terminal lipoylated structure of lipoproteins (Nguyen and Götz, 2016), formylated peptides (Krepel and Wang, 2019), and unmethylated CpG sequences in DNA (Hemmi et al., 2000). This gradient of chemo-attractants induces proinflammatory signaling and activation of local immune response by recruitment of neutrophils and macrophages to the site of infection *via* a process called chemotaxis (Kolaczkowska and Kubes, 2013). Moreover, PSMs secreted by *S. aureus* sheds lipoproteins that also act as neutrophil chemo-attractants (Hashimoto et al., 2006; Hanzelmann et al., 2016). These structures collectively termed as “pathogen-associated molecular patterns” (PAMPs) are specific to bacteria and hence are recognized by the host immune system which activates Toll-like receptors (TLRs; Kumar et al., 2011).

A fibrin pseudo-capsule termed as abscess is formed within 2–6 days of infection (Kobayashi et al., 2015) with the help of coagulase proteins secreted by *S. aureus* which surround bacterial cells and recruited polymorphonuclear leukocytes (PMNs). After recruitment, *S. aureus* further inhibits neutrophil extravasation, activation, and chemotaxis in various ways. The binding of the members of the staphylococcal superantigen-like protein (SSL) family *viz*. SSL2 and SSL4 to TLR2, SSL10 to CXCR4, and SSL5 to GPCRs, such as P-selectin glycoprotein ligand- 1 (PSGL-1; which normally binds to the P-selectin anchor on endothelial cells) on the leukocyte surface, subsequently blocking neutrophil adhesion and extravasation to the site of infection. SelX protein has also been reported to have a similar function as SSL5 (Cheung et al., 2021). CHIP (chemotaxis inhibitory protein) blocks neutrophil recognition of chemotactic factors while Eap (extracellular adhesion protein) prevents neutrophil binding to endothelial adhesion molecule (ICAM-1; Chavakis et al., 2002; de Haas et al., 2004). *S. aureus* protease staphopain degrades CXCR2 (which recognizes cytokines), leading to the inhibition of neutrophil migration toward cytokines (Laarman et al., 2012). Another Geh lipase has been reported that removes the pro-inflammatory lipoylated N- terminus of the bacterial lipoproteins, thereby disguising these PAMPs from neutrophils (Chen and Alonzo, 2019).

Phagocytosis is also inhibited by biofilm formation *via* PIA/PNAG production (refer Section 2.1.1), protective surface structures such as capsules (refer Section 2.1.1), and aggregation. The combined non-redundant activity of coagulase and von Willebrand factor-binding protein (vWbp) of *S. aureus* produces a protein called thrombi which binds to prothrombin (factor II of the coagulation process) and forms a complex called staphylothrombin (Friedrich et al., 2003; Cheng et al., 2010). In the absence of a vascular damage signal, staphylothrombin cleaves fibrinogen from the host cells and forms fibrin clots. Fibrinogen-binding proteins of *S. aureus* such as clumping factor A (ClfA) bind to these clots resulting in the formation of fibrin-containing bacterial aggregates (McAdow et al., 2011). FnBPA and FnBPB can also activate the aggregation of platelets (Fitzgerald et al., 2006). *S. aureus* cells that are already phagocytosed by PMNs fight for their survival by releasing cytolytic toxins which cause pore formation and eventually cell lysis (Figure 1; Peschel and Sahl, 2006; Chambers and DeLeo, 2009; Liu, 2009; Löffler et al., 2010; Spaan et al., 2013; Stapels et al., 2014).

The expression of the capsule, clumping factor A, Protein A, and various other complement inhibitors on the cell surface of the bacterium may help to overcome opsonophagocytosis, i.e., the marking of the bacterial pathogen by antibodies (immunoglobulins, Igs) or complement factors for efficient phagocytosis. The Igs bind to phagocytes by the F_c_ region and to the pathogen by the F_ab_ region. The presence of Igs on the bacterial surface not only marks it for opsonization but also activates the classical pathway of complement fixation (Cheung et al., 2021). *S. aureus* produces three proteins to overcome opsonization by antibodies. (1) Surface protein A (SpA) non-specifically binds to the F_c_ region of IgG (Forsgren and Sjöquist, 1966) and F_ab_ region of IgM, which acts as a B cell superantigen and causes B cell apoptosis. It also initiates the production of plasma B cells that specifically recognizes only protein A, thereby diverting the immune response away from other virulence factors (Goodyear and Silverman, 2003; Pauli et al., 2014). (2) Sbi (*S. aureus* binder of IgG) binds to the complement factor H and C3 apart from the F_c_ region of IgG (Zhang et al., 1998; Atkins et al., 2008). (3) SSL10 also binds to the F_c_ region of IgG and prevents receptor-mediated phagocytosis (Itoh et al., 2010).

All three pathways (lectin, classical, and alternative) of the complement system possess the C3 convertase which cleaves C3 into C3a and C3b. The deposition of C3b on the bacteria marks it for opsonization. Other complement factors such as C5a (formed *via* the interaction of C3-C3b) act as a chemoattractant for the recruitment of more immune cells to the site of infection (Rooijakkers et al., 2005). Staphylococcal complement inhibitor (SCIN) inhibits C3 convertase, thus reducing the C3b deposition and C5a chemoattractant formation leading to the blockage of all three pathways of the complement system. Extracellular fibrinogen binding protein (Efb) of *S. aureus* binds to the C3 component *via* the C-terminus and fibrinogen *via* its N-terminus, covering the bacteria in a fibrinogen layer that inhibits the activation of the complement system as it fails to sense the surface-bound C3b (Ko et al., 2013). A homologous protein of Efb i.e., extracellular complement binding protein (Ecb), although lacking the fibrinogen binding activity, can inhibit the C3 convertase of the alternative pathway and all C5 convertases (Jongerius et al., 2010). Some *S. aureus* proteins such as collagen adhesion protein (Cna) inhibit the classical pathway (Kang et al., 2013), SdrE protein inhibits the alternative pathway (Sharp et al., 2012; Zhang et al., 2017) and the extracellular adherence protein (Eap) inhibits both lectin and classical pathways (Woehl et al., 2014). Finally, the SSL7 protein binds to the C5 component of the complement system as well as to the F_c_ region of the IgA antibody, inhibiting its recognition (Langley et al., 2005).

Proteolytic activity of various *S. aureus* proteins such as staphylococcal serine protease (V8 protease; SspA), cysteine protease (SspB), metalloprotease (aureolysin; Aur), and staphopain (Scp) have also been reported to inhibit opsonization (Dubin, 2002). Though the primary function of these proteases is nutrient acquisition, they may also destroy various immune defense proteins such as aureolysin cleaves C3 (Laarman et al., 2011) and V8 protease cleaves Igs (Rousseaux et al., 1983).

Despite the several mechanisms to evade phagocytosis, neutrophils can still manage to engulf *S. aureus* cells. Primary granules of neutrophil synthesize the enzyme Myeloperoxidase (MPO) which produces reactive oxygen species (ROS) and antimicrobial peptides (AMPs) such as defensins. Secondary granules of neutrophil secrete antimicrobial proteins such as lysozyme. *S. aureus* has evolved with various mechanisms to overcome both ROS and AMPs (Cheung et al., 2021). The orange pigment that gives *S. aureus* (*aureus* stands for golden) its name, Staphyloxanthin has been reported to scavenge the free radicals originating from ROS activity (Pelz et al., 2005; Clauditz et al., 2006). *S. aureus* synthesizes the enzyme superoxide dismutase which converts superoxide to less toxic H_2_O_2_ (Mandell, 1975; Clements et al., 1999). Furthermore, the catalase KatA and alkyl hydroperoxide reductase C AhpC detoxify the H_2_O_2_ by converting it into oxygen and water (Mandell, 1975; Cosgrove et al., 2007). MPO is directly inhibited by a staphylococcal peroxidase inhibitor (SPIN; De Jong et al., 2017).

Defensins and other AMPs are usually positively charged and hence are attracted to the negatively charged cell membrane and exhibit their bactericidal activity by forming pores in the bacterial membrane (Joo and Otto, 2015). The *dlt* operon of *S. aureus* esterifies hydroxyl groups in teichoic acids with alanyl residues which imparts an extra positive charge per alanine into the bacterial cell membrane, increasing the net charge and thereby inhibiting the binding of AMPs (Peschel et al., 1999). Moreover, a membrane-bound enzyme MrpF (multiple peptide resistance factor) adds Lys-PG on the outer layer of the cell membrane which also decreases the affinity of AMP binding (Peschel et al., 2001; Ernst et al., 2015). Finally, *S. aureus* secretes an enzyme named OatA, which acetylates the muramic acid residues of peptidoglycan reducing the efficacy of the neutrophil secreted antibacterial protein lysozyme which is otherwise very effective against other Gram-positive bacteria (Bera et al., 2005).

Neutrophil extracellular traps (NETs) are considered to be an integral component of the human immune system as they play a major role in host defenses during bacterial infections. NETs consist of activated neutrophils and DNA backbone along with proteins of various biological functions. NETs can ensnare but not kill *S. aureus*. The trapped bacteria from NETs can be released by degradation of the DNA backbone *via S. aureus* DNase leading to the persistence of the chronic infection. Eap, a protein secreted by *S. aureus*, can bind and aggregate linearized DNA, hindering the formation of NETs. Apart from this, the pathogen overcomes NET-mediated killing by expressing the surface protein FnBPB, which can neutralize the bactericidal activity of histones (Speziale and Pietrocola, 2021).

When encountered by neutrophils, *S. aureus* biofilms release the leukocidins such as LukAB and PVL (Panton-Valentine Leukocidin), which not only promotes *S. aureus* survival during phagocytosis but also induces the formation of NETs. Neutrophils and NETs can penetrate *S. aureus* biofilm but are unable to disrupt it (Malachowa et al., 2013). NETs can accumulate in organs and can cause tissue damage, as the infection progresses (Saffarzadeh et al., 2012; Weber, 2015). Therefore, the induction in the formation of NETs rather than blocking its antibacterial activity benefits the bacteria by favoring the colonization of deeper tissues, and thus providing better access to metabolic resources. This ensures the safer and optimal survival of bacteria in the host (Speziale and Pietrocola, 2021).

The abscess initially formed and matured within 6–14 days of infection accompanied by fibroblastic proliferation and tissue repair at the abscess margin and the formation of a fibrous capsule at the periphery (Kobayashi et al., 2015). The disruption of abscesses leads to the spread of *S. aureus* beneath the skin surface as well as in the bloodstream causing bacteremia. The bacterium can now adhere to endothelial surfaces and platelets causing endocarditis, metastatic abscesses, and bacterial uptake into endothelial cells where antibiotics and host defense molecules struggle to reach (Chavakis et al., 2005; Weidenmaier et al., 2005; Löffler et al., 2014). If endovascular spread cannot be controlled then systemic blood coagulation, massive production of microorganism-associated molecular pattern molecules (MAMP) and superantigen toxin-mediated cytokine storms can result in systemic inflammation, sepsis, multiple organ failure, and eventually death (Thomer et al., 2016; Lee et al., 2018).

## 3. Virulence factors involved in pathogenesis

*S. aureus* secretes a diverse array of virulence factors such as MSCRAMMs, hemolysins, leukotoxins, Protein A, exfoliative toxins, staphylococcal enterotoxins (SEs), and toxic-shock syndrome toxin-1 (TSST-1; refer Sections 3.1–3.6). Genes encoding these virulence factors (except MSCRAMMs) are located on the accessory genome, which comprises of mobile genetic elements (MGEs) such as plasmids, insertion sequences, pathogenicity islands, transposons, and prophages. These MGEs not only encode the genes for virulence factors production but also contain antibiotic-resistance determinants. Plasmids and transposons contain antibiotic-resistance genes while prophages and pathogenicity islands consist of toxins and other virulence determinants (Malachowa and DeLeo, 2010; Lindsay, 2019). The large family of staphylococcal pathogenicity islands (SaPIs) is widely known for enterotoxins and TSST. Toxins such as staphylococcal superantigen-like genes (SSLs), lipoprotein-like toxins (LPLs), α toxin, PSM peptides, leukocidin LukDE, and some enterotoxins are encoded on genomic islands and vary in their expression among different *S. aureus* isolates (Langley et al., 2010; Malachowa and DeLeo, 2010; Nguyen et al., 2015). Some other *S. aureus* toxins such as PVL, exfoliative toxins A and B, staphylokinase, immune evasion proteins such as CHIPS and SCIN, and several other enterotoxins are encoded on prophages (Cheung et al., 2021). β toxin encoding gene *hlb* has been reported to be non-functional in many *S. aureus* strains by the insertion of phage-encoding genes for CHIPS, SCIN, and staphylokinase (Carroll et al., 1993). This process is termed as “negative conversion” and it can be repaired by phage excision, which is important for infectious colonization (Katayama et al., 2013). Genomic islands such as vSAα, vSAβ, and vSAγ have also been reported to encode a diverse array of virulence factors. These MGEs have lost their ability to be transferred by non-MGE-specific mode of transfer and hence are now very stable and in fact are the characteristic of the entire species (Kläui et al., 2019). The genes encoding MSCRAMMs are located on the core genome, which is evident as they also exhibit general functions in the commensal lifestyle of *S. aureus* (Cheung et al., 2021).

### 3.1. MSCRAMMs

*S. aureus* MSCRAMMs can bind to various proteins present in the extracellular matrix (ECM) of the host. Fibronectin-binding proteins, FnbPA and FnbPB, are responsible for bacterial attachment to fibronectin *in vitro* as well as binding to foreign bodies and plasma clots (Nizet and Bradley, 2011). Fibrinogen binding proteins or clumping factors, i.e., ClfA and ClfB (Ní Eidhin et al., 1998; O'Connell et al., 1998), are responsible for firm attachment of *S. aureus* to vascular thrombi in the situation of flow stress within the bloodstream (Fowler et al., 2000). Endocarditis studies in rats have reported reduced virulence in ClfA mutant *S. aureus* strains (Moreillon et al., 1995). Collagen-binding protein Cna is responsible for adherence to collagenous tissues such as cartilages (Patti et al., 1992). A Cna null mutant strain of *S. aureus* showed attenuated virulence in a murine septic arthritis model (Patti et al., 1994). Other important MSCRAMMs have been discussed in Table 1.

### 3.2. Hemolysins

Hemolysins are toxins that lyse red blood cells as well as immune cells by binding to their specific receptors. Major classes of hemolysins include α, β, and γ hemolysins which are under the regulation of *agr* and *sae* locus. δ hemolysin (under the regulation of *agr* locus), also classified as Phenol Soluble Modulins (PSM), does not require a receptor to exhibit its hemolytic activity (Kong et al., 2016). α hemolysin/alpha toxin, encoded by the *hla* gene, is one of the major toxins under *agr* regulation. The alpha toxin, a 319 amino acid long pore-forming toxin, is shaped like a beta-barrel that can bind to disintegrin and metalloprotease 10 (ADAM10) receptor present on the cell membrane of the human host cell. α toxin can lyse red blood cells and leukocytes but is unable to neutralize neutrophils (Valeva et al., 1997). In animal models, *hla* mutant strains have been reported to cause less disease severity than the wild-type strains, suggesting the importance of hla toxin in staphylococcal infections (Wilke and Wardenburg, 2010; Berube and Bubeck Wardenburg, 2013). β hemolysin is a non-pore-forming toxin that can hydrolyze sphingomyelin, lyse erythrocytes (at low temperature), and monocytes but not lymphocytes and granulocytes (Walev et al., 1996). Its mode of action is still unclear, but it has been hypothesized that as the toxin acts as a sphingomyelinase, it may destabilize the bilipid layer of the plasma membrane of host cells, leading to an alteration in plasma membrane fluidity (Vandenesch et al., 2012). γ hemolysin is a bi-component toxin consisting of two polypeptide chains namely S (slow, HlgA, or HlgC) and F (fast, HlgB). The F component binds to the phosphatidylcholine of host cells, whereas the S component causes cell lysis by binding to the cell membrane of host cells (Meyer et al., 2009). Vandenesch et al. (2012) have reported the lysis of rabbit RBC as well as leukocytes such as macrophages, neutrophils, monocytes, and granulocytes by γ hemolysin. δ hemolysin/phenol soluble modulins (PSMs) are the only peptide toxins of *S. aureus* whose expression is under the direct control of AgrA and independent of RNAIII (Queck et al., 2008). δ toxin is small, amphipathic, and possesses a high affinity to lipids (Vandenesch et al., 2012). These multifunctional peptides produced by many *S. aureus* strains are hemolytic to erythrocytes, various organelles, bacterial protoplasts, and spheroplasts (Verdon et al., 2009). PSMα is a strong pro-inflammatory toxin that can lyse neutrophils post-phagocytosis and also contributes majorly to biofilm formation (Otto, 2014).

### 3.3. Leukotoxins/leukocidins

Leukotoxins such as LukDE, LukGH (LukAB), and Panton-Valentine Leukocidin (PVL) are pore-forming toxins under the regulation of *sae* and *agr* locus (Queck et al., 2008; Cheung et al., 2011; Alonzo and Torres, 2014). Alonzo et al. (2013) have reported lysis of phagocytic cells such as macrophages, dendritic cells, and lymphocytes by binding of LukDE toxin on the CCR5 chemokine receptor present in these immune cells. LukGH (LukAB), similar to PSMα peptide, binds to CD11b receptor in humans and causes lysis of immune cells after phagocytosis (DuMont et al., 2013). Panton-Valentine Leukocidin (PVL), commonly found in community-acquired methicillin-resistant *Staphylococcus aureus* (CA-MRSA), is a two-component toxin (LukS and LukF proteins that binds to TLR2 and TLR4, respectively, in animal models, and C5aR and C5L2 in humans) responsible for causing pore formation in the leukocyte cell membrane, which eventually results into cell lysis and tissue necrosis followed by skin and soft tissue infections (SSTIs; Rasigade et al., 2010). PVL has been reported to be 100-fold more potent than other leukotoxins (Kong et al., 2016).

### 3.4. Protein A

Protein A (*spa*) is one of the major proteases among the *agr* downregulated surface proteins (Cheung et al., 2011). It can activate the TNFα receptor (TNFR1) in the lung parenchyma, resulting in a pro-inflammatory response (Gómez et al., 2004). Once released from the *S. aureus* envelope, Protein A can combat the host's humoral immune response by blocking Fc receptor-mediated opsonophagocytosis and may trigger apoptosis (refer Section 2.1.2; Peterson et al., 1977; Goodyear et al., 2006; Pauli et al., 2014; Le and Otto, 2015).

### 3.5. Exfoliative toxins (ETs)

ETs are serine proteases (under the regulation of *agr* locus) that cleaves the protein desmoglein 1, which leads to the disruption of desmosomal cell linkages causing the detachment of skin epidermis, which results in a surge of infection (Eyre and Stanley, 1987; Hanakawa et al., 2002). ETs are also superantigens but less potent compared to other superantigens such as TSST-1 (Monday et al., 1999). ETs are responsible for a disease named staphylococcal scaled skin syndrome (SSSS), which majorly infects neonates and infants; however, adults who are immunocompromised or have renal impairment are also prone to it. The major symptoms include blistering of the skin, loss of superficial skin layers, dehydration, and other secondary infections (Bukowski et al., 2010).

### 3.6. Staphylococcal enterotoxins (SEs) and toxic-shock syndrome toxin-1 (TSST-1)

Staphylococcal enterotoxins (SEs), secreted by entero-toxigenic *S. aureus* strains, are one of the most common causes of foodborne diseases as these toxins are heat stable. Based on antigenic heterogeneity, there are more than 24 different SEs identified that are under the regulation of *agr* and *sae* locus (Grispoldi et al., 2021). SEA to SEE, SEG to SEI, and SER to SET have reported emetic activity, while staphylococcal-like (SE*l*) proteins (SE*l*L and SE*l*Q) are not emetic in animal models, whereas SE*l*J, SE*l*K, SE*l*M to SE*l*P, SE*l*U, SE*l*U2, and SE*l*V are yet to be tested (Argudín et al., 2010). Ses induce cytokine release by T-cell activation and proliferation causing cell death *via* apoptosis and toxic shock syndrome (Balaban and Rasooly, 2000; Lin et al., 2010). SEF has been renamed as toxic shock syndrome toxin (TSST-1), widely known as a superantigen that can bind to HLA class II molecule on antigen-presenting cells and on T cell receptors leading to massive T-cell activation, proliferation, and release of cytokines (referred to as “cytokine storm”) *viz*., IL-8, MIP-3, IL-2, and TNFα inducing apoptosis and cell death due to lethal toxic shock. In lethal cases, TSST can lead to severe shock, organ dysfunction, and eventually death (Fraser and Proft, 2008; Otto, 2014; Stach et al., 2014). An epidemic was reported in the US from 2004 to 2014, which was found to be common among women using high-absorbency tampons in menstruation (mTSS). However, the incidence rate of mTSS was significantly reduced after the change in manufacturing and awareness regarding the use of tampons (Sharma et al., 2018). Currently, around 50% of TSS infections (such as skin and soft tissue infections) are due to non-menstrual toxic shock syndrome (nmTSS; Sharma et al., 2019).

## 4. Genetic regulation of pathogenesis

**Figure 2:**
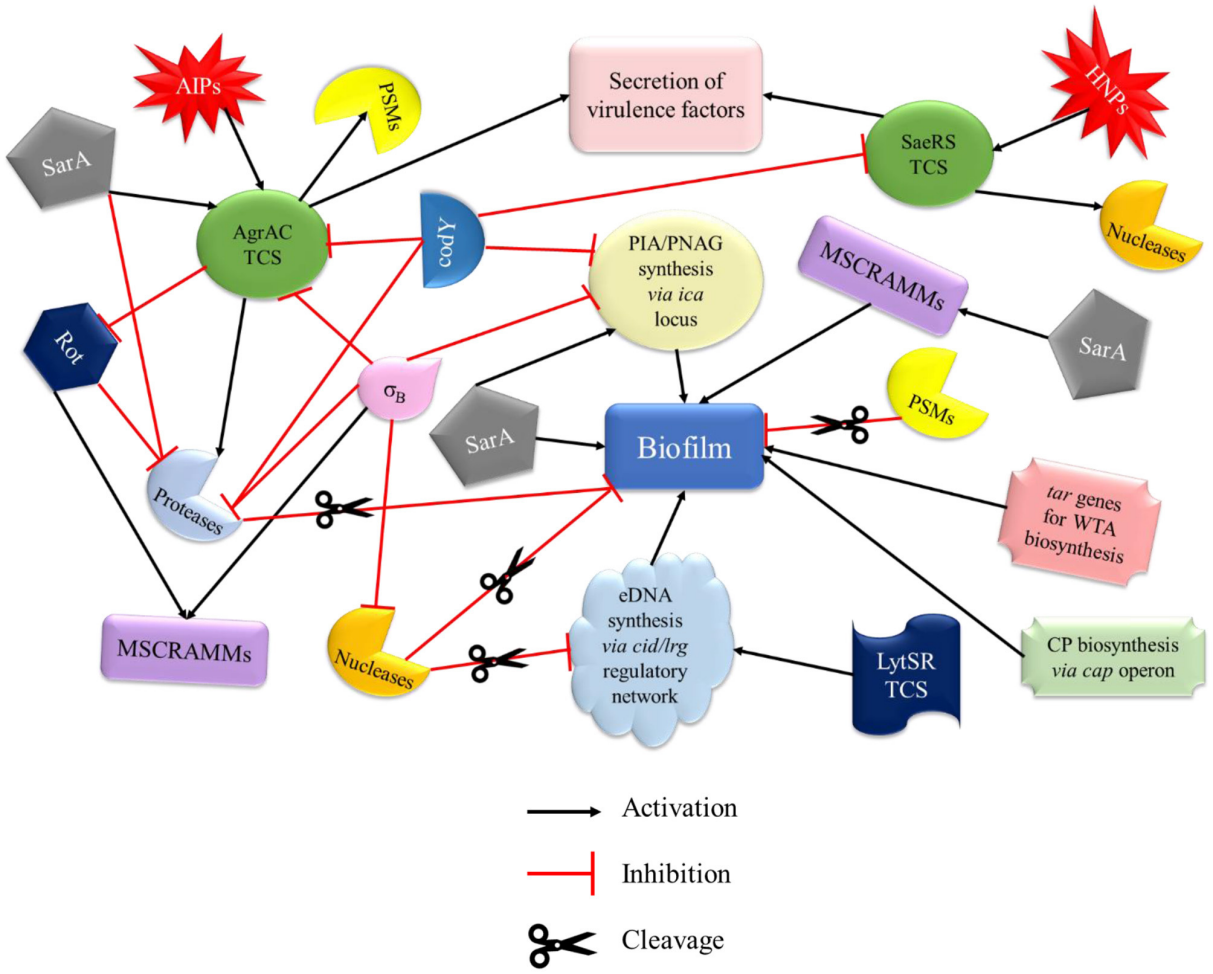
Regulatory network of biofilm and virulence in MRSA. The regulation of biofilm formation and virulence factors of MRSA involves a cross-talk of various systems that drive its pathogenesis. MSCRAMMs, PIA synthesis, CP, WTA biosynthesis genes, and SarA promote biofilm formation. PSMs and proteases (activated by AgrAC TCS) are directly involved in the disruption of biofilm, whereas nucleases (activated by SaeRS TCS and involved in the cleavage of eDNA), σ_B_, and codY (by inhibiting PIA synthesis) play an indirect role. Agr and Sae TCSs, which are mainly involved in the secretion of virulence factors of MRSA, are primarily activated by AIP and HNP, respectively. AgrAC TCS can also be activated by SarA and repressed by σ_B_ and codY. codY is also involved in the downregulation of SaeRS TCS. MSCRAMMs, microbial surface components recognizing adhesive matrix molecules; PIA, polysaccharide intercellular adhesin; CP, capsule; WTA, wall teichoic acid; PSMs, phenol soluble modulins; AIP, autoinducing peptides; HNP, human neutrophil peptides; Rot, repressor of toxins.

**Figure 3:**
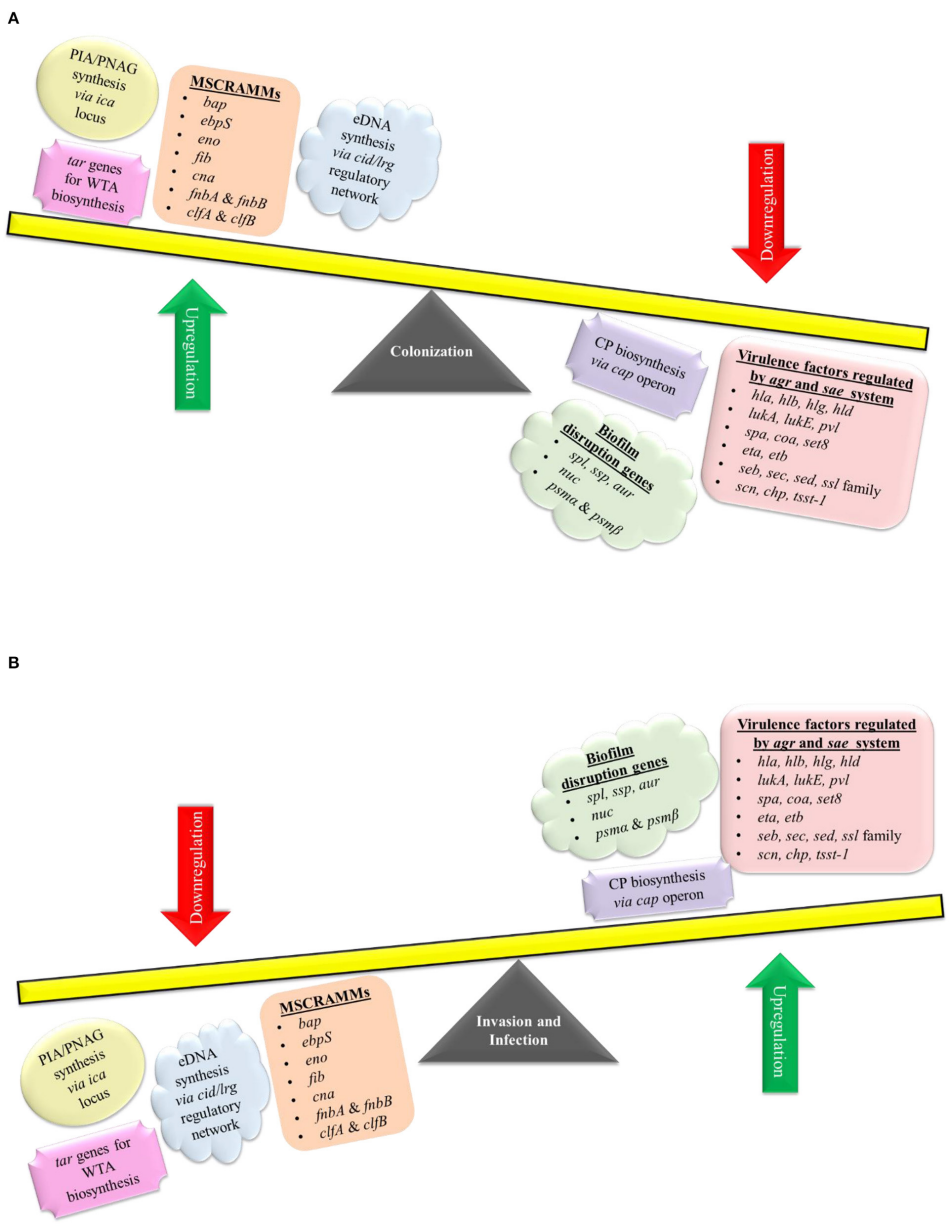
Genetic regulation of pathogenesis. *S. aureus* pathogenesis acts like a see-saw mechanism that involves an up- and downregulation of biofilm and virulence genes during different stages of infection. **(A)** For colonization, *S. aureus* upregulates *ica* locus, *cid/lrg* network, *tar* genes, and MSCRAMMs genes (*bap, ebpS, eno, fib, cna, fnbA, fnbB, clfA*, and *clfB*), which aids in adherence to host tissues as well as biofilm formation. Genes involved in biofilm disruption and virulence factor production are downregulated during colonization. As the infection progresses there is a scarcity of nutrition and oxygen in the matured biofilm, which leads to an **(B)** upregulation of biofilm disruption genes (*spl, ssp, aur, nuc, psm*α, and *psm*β) and genetic systems such as *cap* operon, *agr* & *sae* TCS (which regulates genes such as *hla, hlb, hlg, hld, lukA, lukB, pvl, spa, coa, set8, eta, etb, seb, sec, sed, ssl* family genes, *scn, chp*, and *tsst-1*) that aid in acquiring nutrition, evading immune cells and spread of infection. Genes involved in colonization and biofilm formation are downregulated during the invasion and infection process.

The pathogenesis of *S. aureus* is largely dependent on the formation of biofilm and the production of virulence factors. The genetic regulatory network of biofilm and virulence in *S. aureus* is quite complex including a cross-regulation between different components *viz*. MSCRAMMs*, ica* locus, *cid/lrg* network, *tar* genes, *cap* operon, codY, SarA, and *agr* and *sae* TCS (Figure 2). During the onset of infection, the genetic systems responsible for initial colonization (*viz*. MSCRAMMs genes) and biofilm formation (*viz., ica* locus, *cid/lrg* network, and *tar* genes) are upregulated facilitating adherence to host tissue. As the infection progresses, there is a scarcity of nutrition and oxygen in the matured biofilm (due to an increase in bacterial cell density) that relieves the repression mediated by CodY (a global transcriptional regulator in *S. aureus* which represses the virulence genes with respect to nutrient availability and metabolism). Consequently, there is an upregulation of genetic systems responsible for the production of toxins (*agr* and *sae* TCS) that aid in acquiring nutrition, evading immune cells, and spread of infection. This suggests that apart from regulatory proteins, environmental factors such as nutrition, oxygen, pH, and reactive oxygen species (ROS) can also play a significant role. In this instance, the adhesion factors expressed initially are downregulated leading to the dispersal of biofilm. The dispersed planktonic cells can now adhere and colonize to other sites and spread the disease. The up- and downregulation of adhesion genes involved in biofilm formation and genes responsible for synthesizing virulence factors during different stages of infection act as a genetic regulatory see-saw in the pathogenesis of MRSA (Figure 3).

### 4.1. Regulation of biofilm formation

#### 4.1.1. PIA/PNAG production

The structural proteins, namely IcaA, IcaD, IcaB, and IcaC encoded by the *ica* operon synthesize PIA/PNAG. IcaA and IcaD are transmembrane proteins that simultaneously work as N-acetylglucosaminyltransferase-converting NAG monomers to PNAG oligomers of < 20 residues in length. The membrane-bound IcaC protein transports the PNAG oligomers across the cell membrane. Bacterial cell surface-associated IcaB protein deacetylates the PIA/PNAG oligomers that impart a positive charge to them and thus facilitates binding with the negatively charged bacterial cell surface (Cue et al., 2012).

##### 4.1.1.1. Regulation of *ica* locus

**Figure 4:**
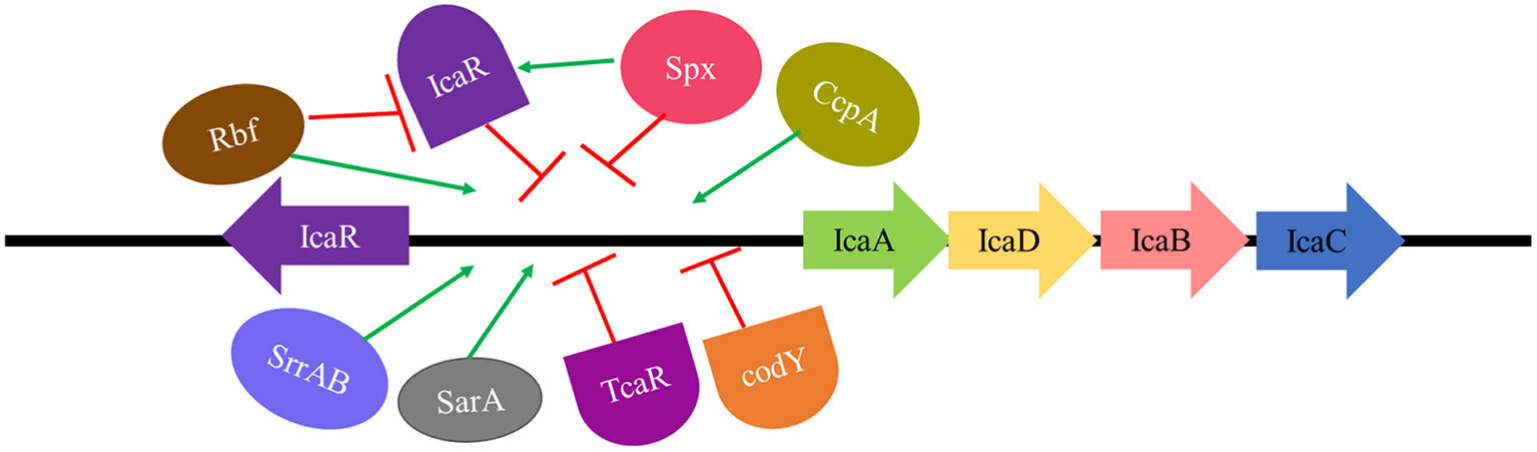
Regulation of *ica* locus. SarA, SrrAB TCS, and CcpA are direct activators, whereas Rbf exhibits an indirect effect in *ica* locus activation by repressing IcaR (repressor of *ica* locus). IcaR, TcaR, and codY are direct inhibitors whereas Spx exerts an indirect effect on *ica* locus repression by activating IcaR (repressor of *ica* locus).

The expression of the *icaADBC* operon is regulated by various direct and indirect factors. SarA and σ_B_ are global regulatory proteins of the operon, whereas some local proteins such as IcaR and TcaR regulate comparatively fewer genes. IcaR directly regulates the *icaADBC* operon, whereas proteins such as Rbf and Spx have an indirect effect (Cue et al., 2012; Figure 4).

(i) **IcaR**

IcaR repressor gene (which encodes the IcaR protein) is transcribed in a divergent manner from the other four genes (Conlon et al., 2002). The binding of IcaR to a DNA region immediately 5′ to the *icaA* gene and deletion of the short nucleotide sequence of 5 bp (TATTT) within the *icaA-icaR* intergenic region could significantly increase the expression of the *icaADBC. icaR* deletion can cause a 100-fold increase in *ica* locus expression and a 10-fold increase in PNAG/PIA production (Jefferson et al., 2004).

(ii) **Sar A**

SarA is a global regulatory protein as it affects the expression of many pathogenic genes in *S. aureus*, hence making it a major virulence factor. SarA protein can function as both activator and repressor of transcription (Bayer et al., 1996; Beenken et al., 2003; Weidenmaier et al., 2005; Oscarsson et al., 2006). SarA can directly bind to the *ica* promoter, enhancing PIA/PNAG production and, subsequently, biofilm formation (Valle et al., 2003; Jefferson et al., 2004; Tormo et al., 2005). The most important role that SarA plays in promoting biofilm formation is the repression of extracellular proteases and nucleases (Schilcher and Horswill, 2020). An elevated expression of proteases was reported in *sarA* mutants (Mrak et al., 2012; Zielinska et al., 2012), which also exhibited a decreased affinity for fibronectin binding (Blevins et al., 2002) and an inability to form static or flow cell biofilm (Beenken et al., 2003). Inhibition of all three classes of proteinases i.e., serine, cysteine, and metalloprotease (Tsang et al., 2008), or concurrent mutation of four extracellular proteases i.e., Aur, ScpA, SspA, and SspB can restore biofilm formation in *sarA* mutants (Loughran et al., 2014).

(iii) **TcaR**

TcaR is a member of the MarR family of transcription factors of *S. aureus*. Jefferson et al. (2004) demonstrated, *via* northern analysis, a 5-fold increase in transcription of the *ica* locus by the inactivation of the *tcaR* gene. The bacterial adherence and PIA/PNAG production were found to increase up to 500-fold in the *icaR tcaR* double mutant, indicating that *tcaR* is a negative regulator of the *ica* locus.

(iv) **σ_B_**

σ_B_ is an alternative sigma factor found in *S. aureus* and other Gram-positive bacteria which is activated by signal transduction in response to environmental stress such as high temperature, extreme pH, high osmolarity, and use of antibiotics (Donegan and Cheung, 2009). Rachid et al. (2000) reported the role of σ_B_ in biofilm formation under high salinity and osmotic stress in an *S. aureus* mucosal isolate. σ_B_ has been reported to have a role in the expression of *ica* operon. Valle et al. (2003) reported that *sarA-* σ_*B*_double mutants showed a decrease in *icaADBC* expression, but, on the other hand, also showed a significant increase in PIA/PNAG production and biofilm formation as compared to single *sarA* mutants. These results suggested the role of σ_B_ in the upregulation of a factor directly involved in the turnover of PIA/PNAG. An enhancement in PIA-dependent biofilm formation has been reported in σ_B_ mutants due to an increased accumulation of IcaC protein (Valle et al., 2019).

The available literature on σ_B_-mediated regulation of PIA-dependent biofilm formation is contradictory. Cerca et al. (2008) studied *icaADBC* and *icaR* expressions in σ_B_ mutant SA113 and Newman strains of *S. aureus*. They concluded that σ_B_ was a positive regulator of both *icaADBC* and *icaR* transcripts. IcaR has been reported to be a weak repressor of *icaADBC* in *S. aureus* strains. Rachid et al. (2000) reported a decrease in the transcription of the *ica* locus in the *sigB* mutant of the MSSA MA12 strain. A biofilm-negative phenotype of the *sigB* mutant SH1000 and USA300 strain LAC (CA-MRSA isolate) was found to have no significant changes in the *ica-* dependent PIA formation but showed an enhanced Agr and protease activity (Lauderdale et al., 2009). However, both these strains were capable of forming PIA-independent biofilms (Boles and Horswill, 2008; Pozzi et al., 2012; Atwood et al., 2015). These contradictory results may be due to variations in some strain-specific properties, and the use of different media as well as biofilm models used in these studies (Schilcher and Horswill, 2020).

(v) **Rbf**

Rbf (regulator of biofilm), a member of the AraC/XylS family, is a transcriptional regulatory protein that plays a critical role in biofilm formation in *S. aureus* (Lim et al., 2004). Rbf positively regulates *icaADBC* transcription by inhibiting *icaR* expression (Gallegos et al., 1997). Cue et al. (2009) analyzed whether the activation of *icaADBC* mediated by Rbf is through direct binding to *ica* promoter or by repression of *icaR*. The results indicated that Rbf was unable to directly bind to the *icaADBC* promoter, suggesting that the expression of *icaADBC* by Rbf is indirect *via* inhibition of the expression of *icaR*.

(vi) **Spx**

Spx protein is a global transcriptional regulator that can act both as an activator as well as a repressor. It blocks transcription by directly binding to the α subunit of RNA polymerase, thereby inhibiting its interactions with target genes (Nakano et al., 2010). Spx mutants were found to exhibit increased transcription of *icaADBC* along with a decrease in *icaR* transcription. Thus, it can be concluded that Spx downregulates *icaADBC* expression by upregulating *icaR* expression. However, the exact mechanism of increased expression of *icaR* by Spx is still unclear (Pamp et al., 2006).

(vii) **SrrAB**

SrrAB is a two-component system (TCS) that prevents the phagocytosis of *S. aureus* by neutrophils in anaerobic conditions which is often found in the core region of a mature biofilm. Ulrich et al. (2007) demonstrated that phosphorylated SrrA protein can bind to 100 bp upstream of the promoter of *ica* operon resulting in an increase in its expression. Under anoxic conditions, the SrrAB mutant showed downregulation of *icaA* transcription and PIA/PNAG production. However, the SrrAB mutation did not exhibit any change in *icaR* expression, suggesting the direct activation of the *icaADBC* by SrrAB.

(viii) **CcpA and CodY**

The expression of *icaADBC* in *S. aureus* is also affected by changes in the levels of the TCA cycle intermediates with respect to the metabolic state of the cell (Vuong et al., 2005). The genes encoding for enzymes involved in the TCA cycle are usually repressed by CcpA in the presence of high glucose concentration among the majority of Gram-positive bacteria including *S. aureus*. High-intracellular levels of glucose-6 phosphate and fructose 1,6 bisphosphate regulate the activity of CcpA, which has been reported to be an activator of *icaADBC*. The synthesis of branched-chain amino acids is repressed due to the downregulation of the TCA cycle in high-glucose conditions. CodY transcriptional regulatory protein, which is responsive to branched-chain amino acids and a repressor of *ica* operon, is also downregulated, resulting in further activation of *icaADBC* and enhanced biofilm formation. When the biofilm matures and nutritional scarcity arises then CcpA activity is repressed, TCA cycle and branched chain amino acid synthesis genes are upregulated, resulting in the activation of *codY* and inhibition of *icaADBC* and, subsequently, biofilm formation. Thus, CodY and CcpA are both regulators of *icaADBC* expression in *S. aureus* (Seidl et al., 2008; Fujita, 2009). Mlynek et al. (2020) suggested that PIA synthesis in cells with low codY activity majorly contributes to biofilm formation. CodY regulates PIA-dependent biofilm formation and *codY* mutant strains exhibit different biofilm phenotypes. For instance, a transposon insertion in the *codY* gene of the clinical isolate S30 revealed reduced biofilm formation and PIA production (Tu Quoc et al., 2007). On the contrary, a *codY* allelic replacement mutation in two *S. aureus* clinical isolates SA564 and UAMS-1, reported an elevated biofilm formation, probably due to higher transcription of *icaA* and increased PIA synthesis during *in vitro* growth (Majerczyk et al., 2008). However, CodY-mediated regulation of the *icaADBC* locus is independent of IcaR (Majerczyk et al., 2008), SarA, and RNAIII (Majerczyk et al., 2010).

#### 4.1.2. cid/lrg system

*cidABC* and *lrgAB* operons of *S. aureus* encodes bacteriophage such as holins and antiholins, respectively (Brunskill and Bayles, 1996b). These operons have also been reported to have a crucial role in staphylococcal murein hydrolase activity (Groicher et al., 2000; Rice et al., 2003). In bacteriophages, holins are responsible for the transport of murein hydrolases across the cytoplasmic membrane for cell lysis and antiholins have an inhibitory activity on the holins (Young, 1992). Similarly, in *S. aureus* the cell lysis and eDNA release are controlled by the antagonistic activity of *cid* and *lrg* gene products. Mutations in either *cid* or *lrg* operons lead to variation in biofilm formation which suggests that fine-tuning between survival and death is essential for a robust biofilm formation (Rice et al., 2007; Mann et al., 2009).

##### 4.1.2.1. Regulation of *cid/lrg* system

**Figure 5:**
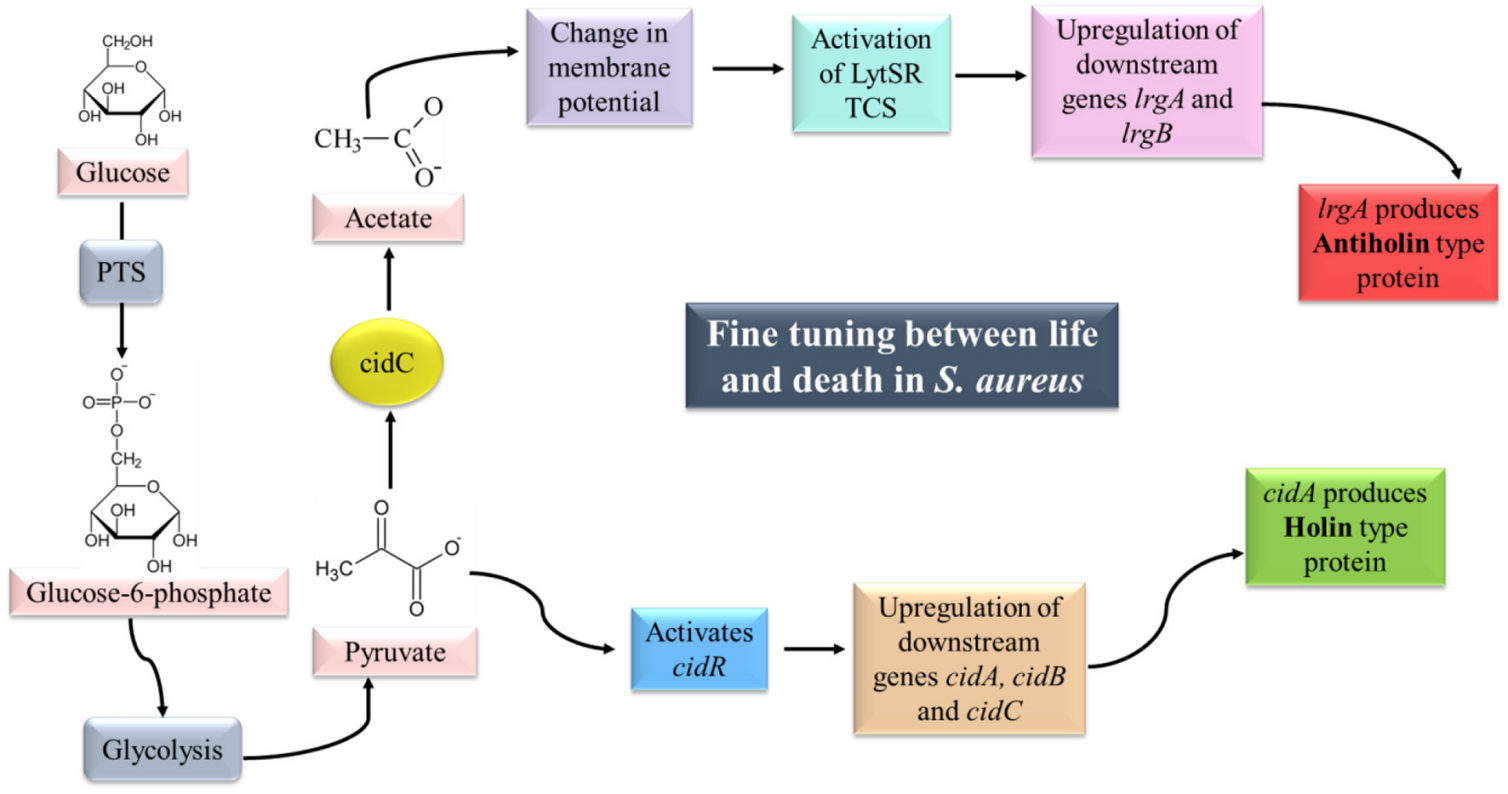
cid/lrg system and its regulation. LytSR TCS is activated by sensing a change in membrane potential (due to the conversion of pyruvate to acetate by CidC). Phosphorylated LytR results in upregulation of the *lrgAB* operon which is responsible for the production of Antiholin-like protein. Pyruvate on the other hand can activate cidR which can upregulate the *cidABC* operon, responsible for the production of Holin**-**like protein. Synthesis of Holin and Antiholin-like proteins maintain a fine tuning between life and death in *S. aureus* (adapted from Bayles, 2007; Sadykov and Bayles, 2012).

The cell death and lysis of *S. aureus* controlled by the *cid/lrg* system are under the regulation of another TCS, i.e., LytSR and transcriptional regulator cidR (Figure 5).

LytSR is a two-component regulatory system composed of a sensory histidine kinase protein (LytS) and a response regulator (LytR), which has been reported to control the expression of *lrgAB* operon (Brunskill and Bayles, 1996a; Sharma-Kuinkel et al., 2009). LytS senses the change in membrane potential of the cell which may be caused due to accumulation of acetate in the media under conditions of excessive glucose and oxygen (overflow metabolism; Rice et al., 2005; Patton et al., 2006). Activated LytS in turn phosphorylates the response regulator LytR, which can induce *lrgAB* expression. Mutation in *lytSR* genes has been reported to cause increased levels of autolysis due to increased murein hydrolase activity (Brunskill and Bayles, 1996a). Hence, LytSR-mediated control over the *lrgAB* operon is essential to regulate the autolysis phenomenon in biofilm.

cidR is a LysR type of transcriptional regulator (LTTR) responsible for DNA binding. Yang et al. (2005) reported two overlapping transcripts (*cidABC* and *cidBC*) of *cid* operon as revealed by northern blot analysis. The transcription of *cidBC* is induced by σ_B_, while the transcription of *cidABC* is dependent on CidR and CcpA (Sadykov et al., 2019). The presence of excess glucose and/or acetic acid induces the transcription of *cidABC*. CidC-encoded pyruvate oxidase converts the end product of glycolysis, i.e., pyruvate into acetate (Patton et al., 2005). Pyruvate is also believed to serve as a co-inducer molecule for the activation of *cidR* (Schell, 1993).

In planktonic cells of *S. aureus*, the expression of *cidABC* is induced under excess glucose leading to a high rate of glycolysis which in turn inhibits aerobic respiration and diverts the carbon flow through fermentation pathways, known as the Crabtree effect. With enhanced murein hydrolase activity due to the activation of *cidABC*, holin-like proteins are produced that aid in the death and lysis of weakened cells. On the other hand, fermentative metabolism and CidC protein lead to the conversion of pyruvate into organic acids such as acetic acid. Acidification leads to disruption in the membrane potential of the cell which is sensed by LytSR TCS that further controls the expression of *lrgAB. lrgAB* transcription leads to the production of antiholin-like protein, thereby maintaining a fine balance between survival and death of *S. aureus* (Sadykov and Bayles, 2012).

The decrease in the expression of *lrgA via* induction of *sigB* by hyperglycemia-related factors such as advanced glycation end products (AGEs) secreted during diabetic foot infection (DFI) in *S. aureus* increases the release of eDNA, thereby enhancing biofilm formation (Xie et al., 2020). σ_B_ has also been reported to repress the expression of the secreted thermonuclease Nuc. Cell-free supernatants of a USA300 *sigB* mutant were found to inhibit the biofilm formation of different *S. aureus* strains. Subsequent fractionation and mass spectroscopy analysis revealed that Nuc was an active component in the supernatant which is responsible for the cleavage of eDNA, subsequently inhibiting biofilm formation. Kiedrowski et al. (2011) reported enhancement in biofilm formation in several *S. aureus* strains, including the USA300 lineage upon deletion of the *nuc* gene. The biofilm-negative phenotype of the *sigB* mutation has been observed to be partially repaired in a *nuc*-*sigB* double mutant.

#### 4.1.3. Capsule biosynthesis

**Figure 6:**
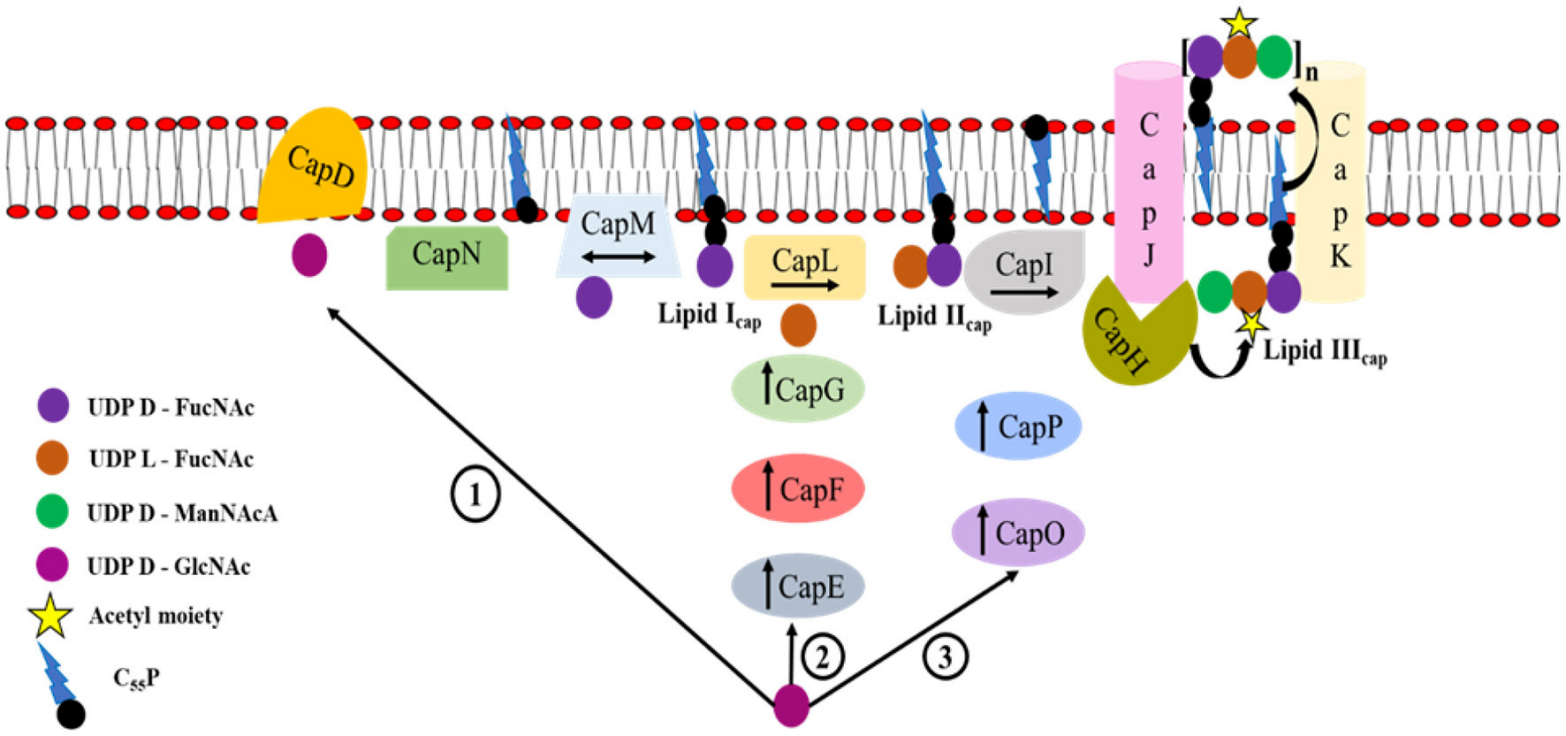
Cascade of capsule biosynthesis. The pathway for capsule (CP) biosynthesis occurs in the cytoplasm *via* three distinct reactions in which the universal cell envelope substrate UDP-D-N-acetylglucosamine (UDP-D-GlcNAc) is converted into the three different nucleotide-coupled sugars: (1) UDP-N-acetyl- D-fucosamine (UDP-D-FucNAc) by the enzymes CapD and CapN, (2) UDP-N-acetyl-L-fucosamine (UDP-L-FucNAc) by the enzymes CapE, CapF, and CapG, and (3) UDP-N-acetyl-D-mannosaminuronic acid (UDP-D-ManNAcA) by the enzymes CapP and CapO. The enzymes CapK and CapJ translocate the modified precursor to the outer surface of the cell membrane where polymerization occurs (modified from Rausch et al., 2019).

CP5 and CP8 capsules (prevalent among *S. aureus* isolates) consist of repeating units of N-acetyl mannosaminuronic acid, N-acetyl L-fucosamine, and N-acetyl D-fucosamine. *S. aureus* have different serotypes due to the difference in glycosidic linkages between the sugars and the sites of O-acetylation of the mannosaminuronic acid residues of the capsule (O'Riordan and Lee, 2004; Kuipers et al., 2016). The pathway for capsule (CP) biosynthesis occurs in the cytoplasm *via* three distinct reactions, in which the universal cell envelope substrate UDP-D-N-acetylglucosamine (UDP-D-GlcNAc) is converted into the three different nucleotide-coupled sugars: UDP-N-acetyl-D-fucosamine (UDP-D-FucNAc), UDP-N-acetyl-L-fucosamine (UDP-L-FucNAc), and UDP-N-acetyl-D-mannosaminuronic acid (UDP-D-ManNAcA; Figure 6; Rausch et al., 2019).

The first reaction is catalyzed by the enzymes CapD and CapN that converts UDP-D-GlcNAc into the first soluble precursor UDP-D-FucNAc. The phosphosugar moiety of UDP-D-FucNAc is transferred to the membrane-anchored lipid carrier undecaprenyl-phosphate (C_55_P) by CapM, yielding lipid I_cap_ (Li W. et al., 2014). The second reaction is catalyzed by the enzymes CapE, CapF, and CapG which convert UDP-D-GlcNAc into a second soluble precursor UDP-L-FucNAc. The transferase CapL further attaches L-FucNAc to lipid I_cap_, resulting in the formation of second CP lipid intermediate, lipid II_cap_ (Kneidinger et al., 2003). The third reaction is catalyzed by the epimerase CapP and the dehydrogenase CapO, which converts UDP-D-GlcNAc into a third soluble precursor UDP-D-ManNAcA (Kiser et al., 1999; Portols et al., 2001). The transmembrane protein CapI transfers the UDP-D-ManNAcA moiety to lipid II_cap_, generating the final capsule precursor lipid III_cap_ (Rausch et al., 2019). The modification of C_55_P coupled trisaccharide is carried out by acetyltransferase CapH, which catalyzes the O- acetylation of L-FucNAc residues at the C3 position in CP5 strains (Bhasin et al., 1998). The putative flippase CapK and the polymerase CapJ translocate the modified precursor to the outer surface of the cell membrane where polymerization occurs (Sau et al., 1997; O'Riordan and Lee, 2004). The attachment of CP precursors to the MurNAc (N-acetylmuramic acid) moiety of peptidoglycan occurs *via* an unknown mechanism that possibly involves a member of the LCP (LytR-CpsA-Psr) family of proteins (Kawai et al., 2011; Chan et al., 2014). This process has been assumed to release the lipid carrier C_55_P, which enters a new synthesis cycle (Rausch et al., 2019).

##### 4.1.3.1. Genetic regulation of capsule expression

*cap5* and *cap8* are allelic gene loci that constitute a 17.5 kb region of the chromosomal DNA. Both loci consist of 16 linked genes [*cap5A (cap8A)* to *cap5P (cap8P)*], which are transcribed in one direction and are involved in biosynthesis, acetylation, transport, and regulation of capsule biosynthesis. In total, 12 out of these 16 genes are nearly identical in both loci (Sau et al., 1997; Rausch et al., 2019). The genes distinguishing CP5 and CP8 strains exhibit very little homology and are located in the central region of the loci (*cap5H, cap5I, cap5J*, and *cap5K* and similarly for *cap8*; O'Riordan and Lee, 2004). Wann et al. (1999) integrated *cap5HIJK* genes into the CP8 strain *via* homologous recombination resulting in a reciprocal loss of *cap8HIJK*. The recombinant strain started producing CP5, indicating that indeed *cap5HIJK* genes were responsible for the CP5 serotype.

*cap8* and *cap5* gene expression are both positively regulated by *agr* locus as well as *sarA*. Single mutants of *agr* and *sarA* as well as *agr-sarA* double mutant studies have reported *agr* locus to be a major regulator of *cap8* gene expression. *sarA* gene was also found to be responsible for the activation of *cap8* gene expression at the transcriptional level but its effect was minor as compared to *agr* (Luong et al., 2002). Positive regulation of *cap5* gene expression by *agr* locus both *in vitro* and *in vivo* was reported in the rabbit endocarditis model. Similar to *cap8* gene regulation, *sarA* was also found to exert a lesser positive impact on *cap5* gene expression (van Wamel et al., 2002). Another global regulator *mgr* belonging to the MarR family of transcriptional regulators has been reported to upregulate CP8 biosynthesis and nuclease expression but downregulated the production of alpha toxin, protease, Protein A, and coagulase (Luong et al., 2003). The global repressor codY has also been reported to repress the *cap* operon under high-nutrient conditions, i.e., early and exponential growth phase (Pohl et al., 2009; Majerczyk et al., 2010). Phosphorylated SaeR (SaeRS TCS) binds to the promoter of the *cap* operon and represses both SigB- and SigA-dependent promoter activities (Keinhoerster et al., 2019). Environmental factors also play a major role in capsule expression both *in vitro* and *in vivo*. High-salt conditions, iron limitation, and growth on solid medium enhance the CP production, whereas high glucose, low oxygen, high CO_2_, alkaline conditions, and yeast extract repress the CP production. CP was reported to rarely express during *ex vivo* analysis of bacteria recovered from cystic fibrosis patients and only a few isolates were CP positive. This might be due to the high CO_2_ concentration in the lungs. Similarly, in cases of nasal colonization also, only a part of the *S. aureus* population was reported to be CP-positive (Keinhoerster et al., 2019).

#### 4.1.4. WTAs biosynthesis

**Figure 7:**
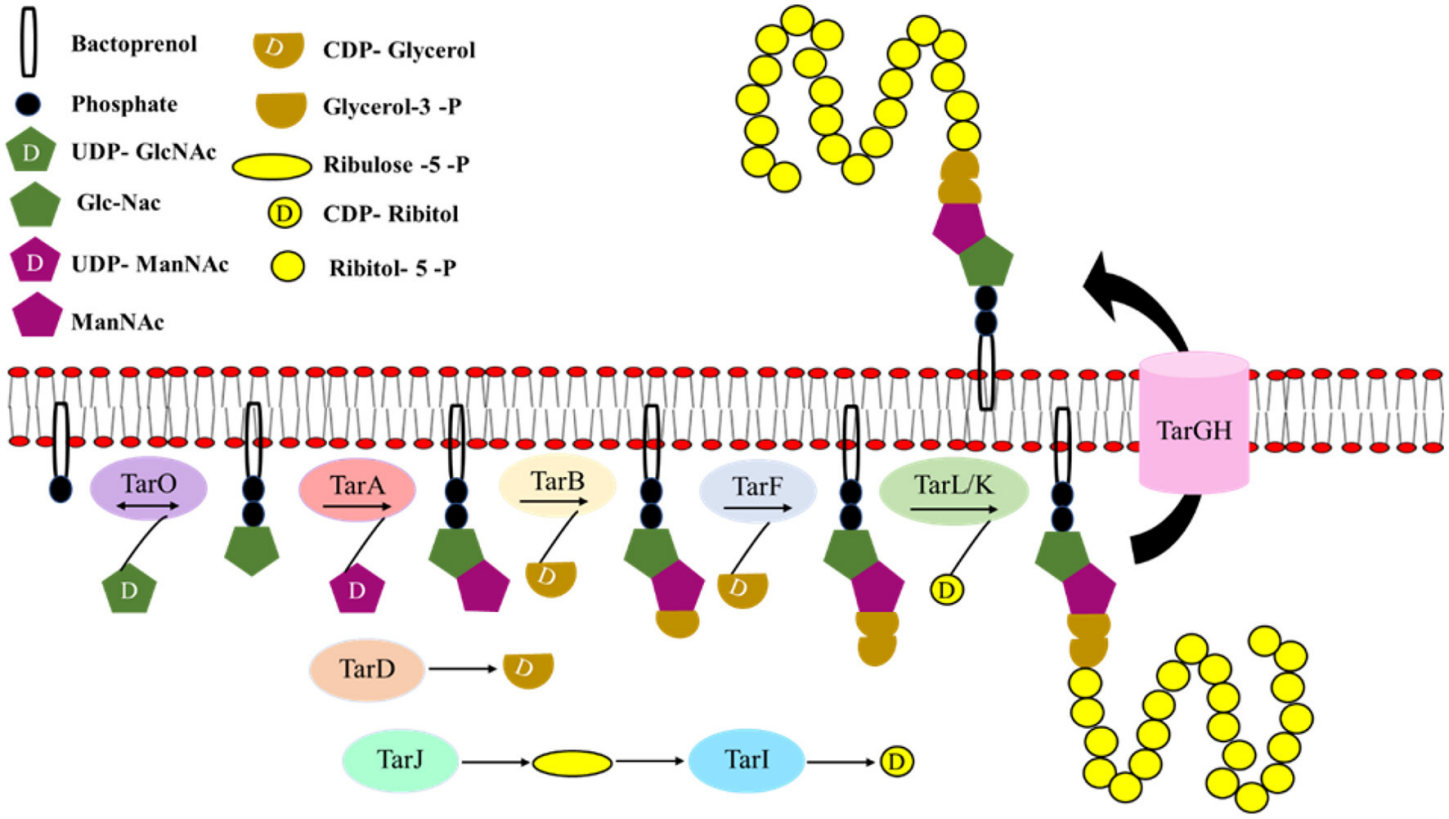
Cascade of wall teichoic acid biosynthesis. The first three steps of the WTA (wall teichoic acid) biosynthetic pathway are catalyzed by the enzymes TagO (TarO), TagA (TarA), and TagB (TarB), respectively leading to the formation of the linkage unit which comprises of a single phosphoglycerol unit from CDP-glycerol to the C4 hydroxyl of ManNAc-β1,4-GlcNAc disaccharide. TarF adds one additional glycerol phosphate unit to the linkage unit. The assembly of the poly(ribitol) phosphate main chain is carried out by one of the two bifunctional poly(ribitol) phosphate primases/polymerases known as TarK/TarL. Once the polymerization is completed in the cytoplasm, glycosylation occurs and the polymer is flipped out by an ABC-dependent transporter complex TarGH followed by which ligation to the cell wall occurs (modified from Swoboda et al., 2010).

WTA biosynthesis was first characterized in *Bacillus subtilis* 168, which makes poly(glycerol) phosphate, and hence the genes involved in its synthesis were known as *tag* genes (for teichoic acid glycerol). *Bacillus subtilis* W23 and *S. aureus* make poly(ribitol) phosphate and hence the genes involved in its synthesis were known as *tar* genes (for teichoic acid ribitol; Ward, 1981; Figure 7).

The first three steps of the biosynthetic pathway are catalyzed by the enzymes TagO (TarO), TagA (TarA), and TagB (TarB), respectively. In the cytoplasm, N-acetylglucosamine phosphate (GlcNAc-1 phosphate) is transferred to an undecaprenyl phosphate (bactoprenyl phosphate) by a reversible phosphosugar transferase enzyme, TagO (TarO; Soldo et al., 2002; Price and Tsvetanova, 2007). Furthermore, TagA (TarA), an N-acetylmannosaminyl transferase, catalyzes the transfer of ManNAc from UDP-ManNAc to the C4 hydroxyl of GlcNAc residue to form ManNAc-β1,4-GlcNAc disaccharide (Ginsberg et al., 2006; Zhang et al., 2006). Finally, TagB (TarB), a glycerophosphotransferase, catalyzes the transfer of a single phosphoglycerol unit from CDP- glycerol (synthesized by TagD or TarD) to the C4 hydroxyl of ManNAc to complete the synthesis of linkage unit (Ginsberg et al., 2006; Bhavsar et al., 2007). The WTA linkage unit is highly conserved in all Gram-positive bacterial strains characterized so far. After these first three initial steps, the WTA pathways diverge (Brown et al., 2013).

In *S. aureus*, TarF (homolog of TagF that acts as a polymerase), which acts as a primase, adds one additional glycerol phosphate unit to the linkage unit (Swoboda et al., 2010). The assembly of the poly(ribitol) phosphate main chain is carried out by one of the two bifunctional poly(ribitol) phosphate primases/polymerases known as TarK/TarL. *S. aureus* TarL is a bifunctional enzyme having both primase and polymerase activities (Meredith et al., 2008). A cytidylyl transferase TarI and an alcohol dehydrogenase TarJ together synthesize the CDP-ribitol substrate that is utilized by TarL. TarL attaches more than 40 ribitol phosphates to complete the polymer synthesis (Brown et al., 2013). *tarK* gene is highly homologous to the *tarL* gene, suggesting that it might have the same enzymatic function. The reason for the presence of two homologous sets of *tarIJL* genes (*tarI'J'K* genes) with the same function (Qian et al., 2006) in *S. aureus* is still not clear; however, Meredith et al. (2008) have reported that *tarK* can nullify the loss of *tarL* and have an enzymatic function similar to *tarL* the gene product. However, strains that produced only TarL or TarK produced two electrophoretically distinct poly (ribitol) phosphate WTAs, known as L-WTA or K-WTA, respectively. K-WTA was found to be significantly shorter than L-WTA based on PAGE analysis. *tarK* gene expression is negatively regulated by *agr* quorum sensing system. As the expression of *tarK* can shorten K-WTA length by 50%, it is presumed that WTA chain length is dynamically regulated by *S. aureus* between pro-adhesion and low adhesion state to promote adhesion and dispersal of biofilm during different stages of infection. Once the polymerization is completed in the cytoplasm, glycosylation occurs and the polymer is flipped out by an ABC-dependent transporter complex TarGH followed by which ligation to the cell wall occurs (Swoboda et al., 2010).

##### 4.1.4.1. Regulation of WTA biosynthesis

Highly pathogenic CA-MRSA strains express the increased level of WTAs by upregulating the TarH ATPase subunit of the TarGH ABC transporter. This “WTA-high phenotype” is expressed in highly virulent strains in which the *agr* quorum sensing system is upregulated (van Dalen et al., 2020). Wanner et al. (2017) have reported increased skin abscess formation, T-cell proliferation, and IFN-γ production in mouse skin models infected with WTA-high phenotype strains. When *agr* is active, L-WTA is expressed resulting in long chains that may aid in colonization and infection (Meredith et al., 2008). Cross-regulation of WTA and CP biosynthesis is another important factor as both processes compete for C_55_P lipid carriers. CP production is thus very tightly regulated and only expressed in post exponential phase that too in a fraction of the population in order to ensure sufficient C_55_P lipid carrier for WTA production which is an inevitable component of the bacterial cell wall (Keinhoerster et al., 2019).

### 4.2. Regulation of virulence factor production

#### 4.2.1. agr locus

**Figure 8:**
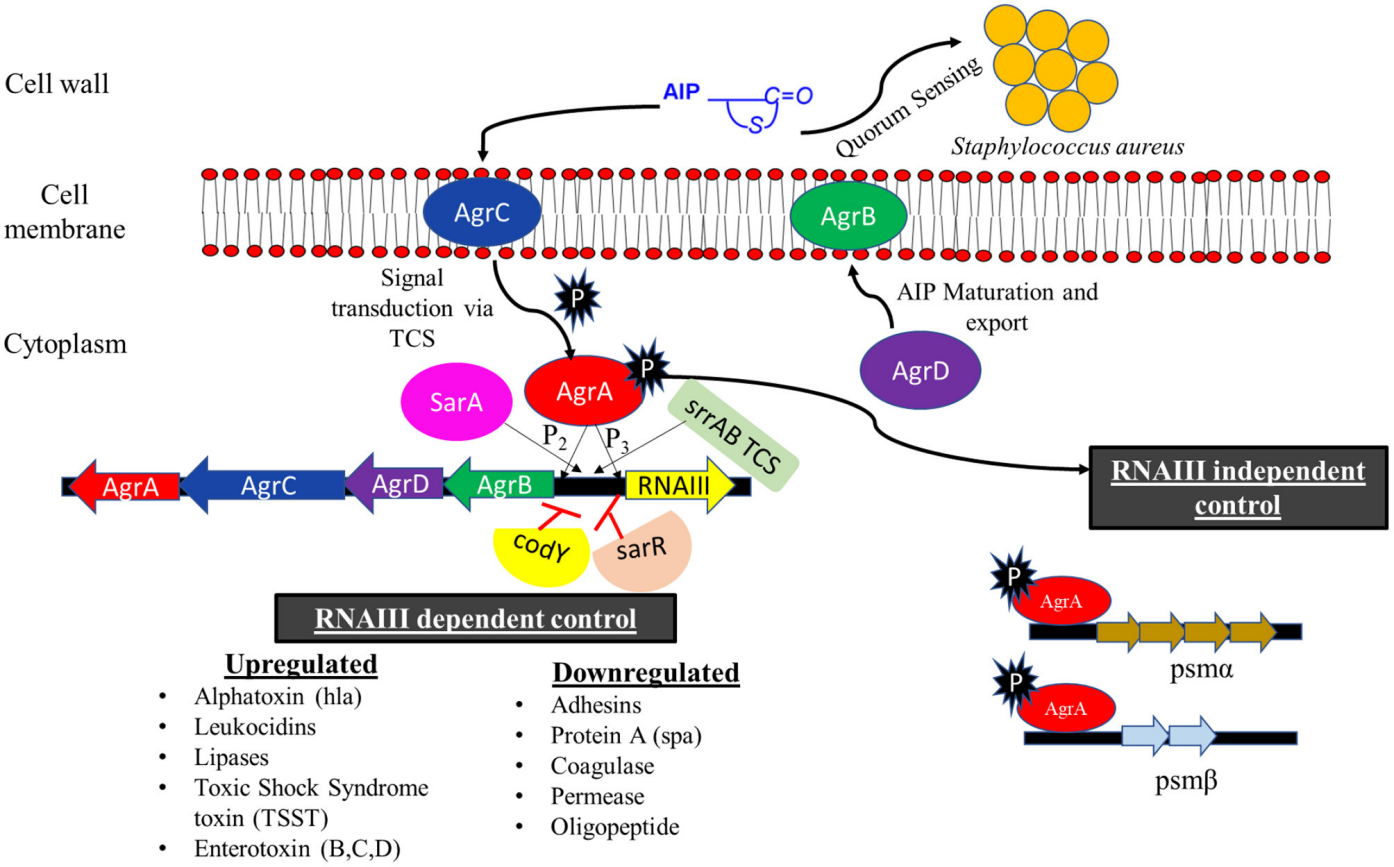
Regulation of *agr* (accessory gene regulator) two-component system. Autoinducing peptide (AIP) synthesized by AgrD is transported across the cell membrane by transmembrane protein AgrB. AIP acts as a signal for quorum sensing in *S. aureus* and can also activate the AgrAC TCS. The activated AgrA can bind to the P2 and P3 promoter of *agr* locus and initiate the transcription of *agrACDB* genes and RNAIII transcript, respectively. RNAIII transcript can mediate the expression of various virulence and biofilm disruption genes. The activated AgrA initiates the transcription of *psm*α and *psm*β in RNAIII independent manner. SarA and SrrAB positively regulate the *agr* locus whereas codY and sarR exhibit a negative effect (modified from Le and Otto, 2015; Tan et al., 2018).

The *agr* locus (first reported by Peng et al., 1988) is 3.5 kb in size and consists of two divergent promoters P2 and P3 that generates the transcripts of RNAII and RNAIII, respectively. RNAII locus comprises of four genes namely, *agrB, agrD, agrC*, and *agrA. agrD* transcript encodes a 7–9 amino acid long autoinducing peptide (AIP), which also plays a role in extracellular quorum sensing signal in *S. aureus* (Ji et al., 1995). AgrB is a multifunctional endopeptidase that is responsible for maturation (thiolactone modification and C terminal cleavage) and the export of AIP across the cell membrane. AgrC and AgrA constitute the two-component signal transduction system, of which AgrC is a membrane-bound histidine kinase, which is auto-phosphorylated upon the binding of AIP. It then trans-phosphorylates the response regulator AgrA. Activated AgrA can bind to the P2 and P3 promoters of *agr* locus and can regulate the expression of downstream genes (Novick et al., 1995; Queck et al., 2008; Le and Otto, 2015; Figure 8).

The majority of the virulence factors that are under the control of *agr* system are regulated by RNAIII. It is a messenger RNA that contains the *hld* gene for delta toxin or delta hemolysin (Janzon et al., 1989). It also activates the transcription of the *hla* gene for an alpha toxin or alpha hemolysin. RNAIII controls the expression of surface proteins, such as Protein A, coagulase, and fibrinogen binding protein, and repressor of toxin (Rot) protein by antisense base pairing with 5′ untranslated regions (5′ UTR) and forming RNA duplexes (Boisset et al., 2007). Rot protein binds to the promoter of many exoproteins (α, β, and γ hemolysin), enterotoxins (Toxic shock syndrome toxin), and leukocidins, inhibiting their transcription. The inhibition of Rot and surface proteins by RNA III leads to an upregulation of virulence factors along with the dispersal of biofilm. AgrA can also upregulate the transcription of phenol-soluble modulins *psm*α and *psm*β operons in an RNAIII-independent manner (Queck et al., 2008; Le and Otto, 2015).

*agr* system is a global regulatory system of *S. aureus* for the upregulation of virulence factors, which aids *S. aureus* to cause several types of infections (Li S. et al., 2014; Tuchscherr and Löffler, 2016) and downregulation of surface proteins for disruption of biofilm. During the initial course of bacterial infection when the cell density is low, *agr* system is downregulated, resulting in an increased production of adhesins and surface proteins. Once colonization is established and nutrients become limited, the upregulation of degradative exoenzymes and toxins mediated by *agr* occurs, which helps not only to acquire nutrition for the cell but also to evade the host immune system (Fowler et al., 2004; Cheung et al., 2014). Yarwood and Schlievert (2003) have reported thicker and smoother biofilms on medical devices formed by *agr* mutant strains, leading to chronic and persisting infections in the host due to their inability to disseminate from the patient's body (Shopsin et al., 2010). The ability of *Staphylococcus aureus* to form biofilm in chronic relapsing infections associated with medical devices such as urinary catheters, intravenous catheters, and orthopedic prosthetics (Singh and Ray, 2014) shields the bacteria from the host immune system as well as antibiotics (Waters et al., 2016).

*S. aureus* strains can be classified into four agr groups (agrI, agrII, agrIII, and agrIV) based on the *agrD* gene (which encodes the synthesis of AIPs) and *agrC* gene (which encodes the receptor of *agr* TCS). The four AIP molecules are similar enough to bind with the AgrC receptor of the different groups but cannot activate the AgrA protein unless it is activated by the AIP of a similar group (Jabbari et al., 2012). Ikonomidis et al. (2009) and Khoramrooz et al. (2016) have reported that strains of *agr* group II and *agr* group III are more potent biofilm producers. Nichol et al. (2011) have also established a relationship between antibiotic resistance and *agr* groups in *S. aureus*. *agr* group I is widely associated with CA-MRSA genotypes, whereas *agr* group II is associated with HA-MRSA genotypes in humans.

##### 4.2.1.1. Regulation of *agr* locus

(i) **SarA family**

It is encoded by the *sar* gene present in the *sar* locus (an important global virulence regulon that plays a major role in growth, biofilm formation, and production of toxins in *S. aureus*). It binds to P2–P3 intragenic region and activates *agr* transcription while another protein Sar R (which also binds to P2-P3 intragenic region) represses the transcription of *agr* (Reyes et al., 2011). *sar* locus possesses ~50,000 copies of the *sarA* gene per cell and it can regulate the expression of nearly 120 genes (encoding virulence, cell wall associated, and extracellular proteins) of *S. aureus* (Cheung et al., 2008; Fujimoto et al., 2009). It consists of three transcripts i.e., *sarA, sarB*, and *sarC* under the control of P1, P2, and P3 promoters, respectively (Cheung and Manna, 2005). SarA is a DNA binding protein that can bind to the P2 promoter of *agr* locus and upregulate the transcription of RNA III. Hence, the *sar* locus can directly activate the *agr-*mediated quorum sensing in *S. aureus via* the P2 promoter of *agr* locus (Rechtin et al., 1999; Sterba et al., 2003; Roberts et al., 2006). α toxin, phenol-soluble modulins, and Panton Valentine Leukocidin (PVL), which are responsible for causing critical infection in CA-MRSA, are regulated by SarA (Bronner et al., 2000; Dumitrescu et al., 2011; Zielinska et al., 2011). Production of extracellular proteases such as serine protease, cysteine protease, metalloprotease, and staphopain are also negatively regulated by SarA (Karlsson and Arvidson, 2002). It can bind to various promoters *viz*., *sarA, mecA*, and *sarR* under the same conditions and at the same time point as revealed by DNA affinity capture assay (DACA; Kim et al., 2022). *Sar* locus has been reported to play a major role in regulating antibiotic resistance mechanisms. Proteomic analysis revealed that *mecA* expression was reduced in *sarA* deficient mutant, the exact reason for which is still unknown. However, this might be a reason for antibiotic susceptibility in *sarA* deficient mutant strains as both genes are involved in biofilm formation and PBP2a expression (Kim et al., 2022). Also, the inactivation of *sarA* has led to a reduction in ciprofloxacin and vancomycin resistance (Lamichhane-Khadka et al., 2009). Sequence analysis of clinical strains of various lineage revealed that the *sarA* gene was highly conserved unlike the other global virulence regulator AgrA which had various mutations (Kim et al., 2022). SarA regulates the post-transcriptional expression of *spa* and collagen adhesion genes during the exponential growth phase by binding to the target mRNA. It changes the turnover as well as accounts for the stability of their transcripts (Roberts et al., 2006; Morrison et al., 2012). Unlike *agr* locus, whose transcription is initiated majorly in the exponential phase, SarA protein levels are constant throughout the growth phases of the bacterium (Cheung and Manna, 2005; Arya and Princy, 2013).

*S. aureus* has various SarA paralogs i.e., SarR, SarS, SarT, Rot, SarU, SarV, SarX, MgrA, and MarR, which are either inhibitor or stimulator of each other, and indirectly contributes to virulence, biofilm production, autolysis, antibiotic resistance, and metabolic processes (Trotonda et al., 2008; Ballal and Manna, 2009). Northern blot and transcriptional fusion analysis have confirmed that SarV, a 116-residue long polypeptide, which is a homolog of SarA and regulator of cell lysis, is regulated by *sarA* as well as *mgrA*. Various virulence and autolysis genes have been reported to be under the regulation of *sarV* (Cheung et al., 2008; Trotonda et al., 2009). SarR, a 13.6 kDa dimeric protein, is a repressor of SarA, which regulates *agr* expression directly by binding to the intragenic region of the P2–P3 promoters, where SarA also binds but with less affinity than SarR. SarR binds to *sarA* or the promoters of target genes, repressing the expression of *sarA, agr, hla, hlb*, and *spa* during the post-exponential growth phase (Cheung and Zhang, 2001; Manna and Cheung, 2001; Oscarsson et al., 2005; Arya and Princy, 2013).

SarS, a 250-residue-long polypeptide with 64% homology with SarA, is a repressor of *hla* transcription and activator of protein A (*spa*; Manna and Ray, 2007). Rot, a 166 amino acid long residue, is an additional regulatory protein that modulates the expression of *sarS*, a transcriptional regulator of virulence genes. Rot protein has been reported to negatively regulate the production of various toxins such as lipases, serine proteases, α toxin, β toxin, cysteine proteases, and several other proteases, suggesting that *rot* acts downstream of the *agr* locus and indirectly upregulates cell wall synthesis. Activation of *agr* results in the production of RNA III which inhibits the production of *rot* (Said-Salim et al., 2003; Schmidt et al., 2003). Treatment with protease inhibitors such as cysteine protease inhibitor E-64 or staphostatin SspC, a specific inhibitor of staphopain B, is necessary to restore biofilm formation in a *rot* mutant (Mootz et al., 2015). SarA and Rot both repress protease production and are thus important regulators of biofilm formation.

SarT, another 118-residue-long homologous protein of SarA that downregulates *hla* and RNA III expression, is negatively regulated by *sarA* (Manna and Cheung, 2003). SarU, a 247- residue-long protein with a molecular mass of 2.92 kDa, is adjoining to *sarT* but is transcribed in the opposite direction. Mutational analysis revealed an elevated expression of *sarU* in *sarT* mutants indicating that *sarU* is negatively regulated by *sarT*. *sarU* mutants exhibited a lower RNAII and RNAIII expression as compared to the parental strain as proved by transcriptional and northern blot analysis. This observation suggests that *sarU* indirectly activates *agr* locus by upregulating RNAIII expression and altering the expression of *agr*-mediated virulence genes (Liu et al., 2006; Arya and Princy, 2013). Finally, the global regulator MgrA (member of the SarA protein family) acts as a repressor of eight cell wall-anchored proteins. *mgrA* mutants exhibited an increased biofilm formation with a loss of bacterial clumping (Schilcher and Horswill, 2020).

(ii) **SrrAB**

SrrAB is a two-component system where SrrA is a 28 kDa, 241 amino acid long response regulator, and SrrB is a 66 kDa, 583 amino acid long histidine kinase. Low oxygen levels and redox environmental conditions such as pH serve as a signal for SrrAB TCS. Upon receiving the signal, the membrane-bound SrrB auto-phosphorylates at a conserved histidine residue. This phosphate group is then transferred to the aspartate residue of cytoplasmic SrrA. SrrA has been reported to bind to both P2 and P3 promoters of *agr* system, positively affecting its activity (Pragman et al., 2004; Tan et al., 2018).

(iii) **CodY**

The nutritional status of the cell greatly impacts biofilm formation and virulence factors production, which is regulated *via* CodY (global transcriptional regulator) in *S. aureus* (Schilcher and Horswill, 2020). CodY has been reported to strongly repress agr locus (Majerczyk et al., 2008) not by binding to the P2 and P3 promoter regions but rather with a region within the *agrC* gene (Majerczyk et al., 2010). However, this had no effect on *agrA* expression. On the contrary, Roux et al. (2014) reported *in vitro* binding of CodY to the P2 and P3 promoter of *agr* locus but with low affinity. Recently, CodY exhibited repression of *rsaD*, a small regulatory RNA (sRNA) responsible for causing cell death regulation during weak acid stress has been reported, which eventually causes eDNA release and biofilm formation (Augagneur et al., 2020).

#### 4.2.2. sae locus

**Figure 9:**
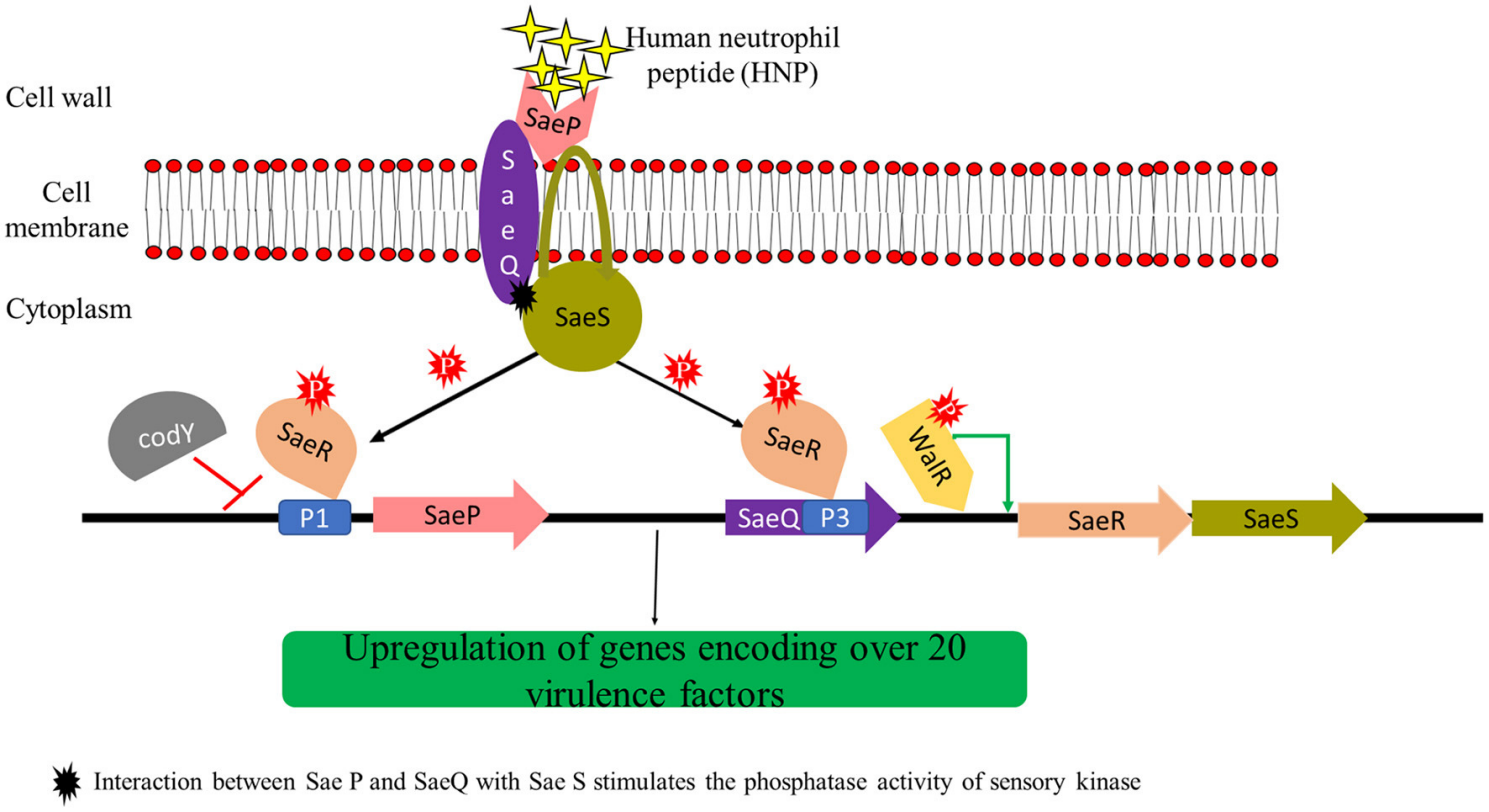
Regulation of *sae* (Staphylococcal accessory element) two-component system. Inducing molecules such as Human Neutrophil peptide 1 (HNP-1) are sensed by the extracellular membrane protein SaeP which interacts with the extracellular linker peptide of the cytoplasmic domain SaeS, activating the SaeRS TCS. Activated SaeR can induce the transcription of *saePQRS* (and genes encoding over 20 virulence factors) *via* P1 and P3 promoters. SaeP and SaeQ can form a protein complex with SaeS and induces the sensory kinase phosphatase activity resulting into the dephosphorylation of activated SaeR leading to the downregulation of virulence genes which are regulated by *saeR*. All these interactions are based on predictions (modified from Liu et al., 2016).

The *sae* (staphylococcal accessory element) system was first identified by Giraudo et al. (1994) while studying a Tn551 insertional mutant in which the production of exoproteins such as nuclease, coagulase, α hemolysin, and β hemolysin was found to be altered. The *sae* operon consists of four genes, i.e., *saeP, saeQ, saeR*, and *saeS*, which are under the regulation of P1 and P3 promoters. The *sae* locus primarily comprises of a two-component system in which SaeR is the response regulator and SaeS is the histidine kinase (Giraudo et al., 1999). This system is activated by external stimuli such as H_2_O_2_ and α defensins and repressed under low pH and high NaCl concentrations, both resulting in membrane perturbation (Geiger et al., 2012; Haag and Bagnoli, 2015). The signal is sensed by SaeP which interacts with the extracellular linker peptide of SaeS leading to its autophosphorylation at His131 which then transphosphorylates SaeR at Asp51. SaeR can now bind to SBS (SaeR binding sequence), leading to the upregulation of *saeR-*mediated virulence genes (Figure 9).

Very little was known about the role of auxiliary proteins SaeP and SaeQ in Sae TCS activity and disease progression. Collins et al. (2020) generated mutants of *saeP, saeQ*, and *saeP-saeQ* double mutant of the USA300 strain of *S. aureus* to analyze their function. The survival of *S. aureus* USA300Δ*saeP* increased compared to the wild type USA300 strain post phagocytosis by neutrophils probably due to increased expression of bicomponent leukocidins by the former. The USA300Δ*saeQ* also yielded a similar phenotype but the neutrophil interaction results were comparatively less significant. However, the *saeP-saeQ* double mutant strain (USA300Δ*saePsaeQ*) exhibited a drastic increase in neutrophil survival and virulence during murine bacteremia as compared to the USA300Δ*saeP* single mutant strain. These observations suggested the role of SaeP in the survival of bacterium after phagocytosis by neutrophils and the combined effect of SaeP and SaeQ in *in vivo* pathogenesis.

Flack et al. (2014) have reported that SaeS more specifically detects α-defensin 1/Human neutrophil peptide 1 (HNP-1) and human polymorphonuclear leucocytes (PMNs). SaeR and SaeS are transcribed *via* the P3 promoter located within the *saeQ* coding sequence. The P1 promoter is located upstream of the *saeP* gene and can transcribe all four genes of the locus. The P1 promoter has been reported to exhibit 2–30 times higher activity compared to the P3 promoter. Jeong et al. (2011) have reported that only basal level expression of *saeRS* genes from the P3 promoter is sufficient for activation of *sae* target genes and exoprotein production. P1 promoter has two *saeR* binding sites and thus it can be auto-induced by *saeR* transcripts produced by basal level expression of *saeRS* TCS *via* P3 promoter (Figure 7). The target genes of the *sae* system have been bifurcated into two classes: Class I target genes require high levels of phosphorylated SaeR for its activation, *viz*., *fnbA, coa*, P1 promoter, *sae*, and *eap*, while Class II target genes require basal/lower levels of phosphorylated SaeR for its activation, *viz*., *hla* and *hlb* genes (Mainiero et al., 2010; Haag and Bagnoli, 2015). SaeP and SaeQ have been reported to form a protein complex with SaeS and induce the sensory kinase phosphatase activity resulting in dephosphorylation of activated SaeR which downregulates the virulence genes regulated by *saeR* (Jeong et al., 2012; Haag and Bagnoli, 2015).

Depending on growth conditions, *sae* system can both positively (upregulation of biofilm forming genes, i.e., *hla, hlb, coa, emp, eap, fnBPA*, and *fnBPB*) and negatively (upregulation of biofilm dispersal factors such as nucleases and proteases) affect biofilm formation (Caiazza and O'Toole, 2003; Johnson et al., 2008; O'Neill et al., 2008; Huseby et al., 2010; McCourt et al., 2014; Zapotoczna et al., 2015; Liu et al., 2016). Moormeier et al. (2014) have reported that *nuc* transcription is positively regulated by the SaeRS system, as both *sae* and *nuc* mutant were unable to cause the dispersal of biofilms, which likely explains the degradation of eDNA during the dispersal stage of biofilm formation. Moreover, SaeS has been reported to exhibit polymorphism across *S. aureus* strains as a point mutation can lead to hyperactivation of SaeRS TCS leading to the inability of such strains to form robust biofilms (Mainiero et al., 2010; Cue et al., 2015). *saeRS* locus also acts synergistically with *sarA* to inhibit the production of extracellular proteases, resulting in an improved ability for biofilm formation (Arya and Princy, 2013).

SaeRS system positively regulates NETs (Berends et al., 2010; McDonald et al., 2012), SCIN, CHIPS (Rooijakkers et al., 2006), leukocidins (Münzenmayer et al., 2016), α hemolysin (Hla; Nygaard et al., 2013), pro-inflammatory cytokines, proteases, and toxins production (Watkins et al., 2011; Zurek et al., 2014; Cho et al., 2015). However, *S. epidermis*, which also possesses a SaeRS two-component system and is closely related to *S. aureus*, does not possess these virulence genes (Handke et al., 2008; Ravcheev et al., 2011). This indicates that *S. aureus* has acquired these virulence genes during evolution, which are being maintained by it under the control of the SaeRS two-component system (Liu et al., 2016).

##### 4.2.2.1. Regulation of *sae* system

Transcription of *sae* operon has been reported to be modulated by other global and local regulatory systems, which are mentioned as follows:

(i) **agr system**

The majority of toxins and exoproteins under the control of *agr* and *sae* operons are similar; however, there are several reports which suggest that *agr* and *sae* are independent of each other (Liu et al., 2016). *sae* operon does not possess AgrA or RNAIII binding sites, indicating that activation of *agr* operon should not have any significant effect on *sae* operon. Some of the virulence genes under the regulation of *agr* and *sae* are transcribed in opposite manner. *coa* and *fnbA* genes are repressed, whereas the *cap* gene is activated by *agr* operon. On the other hand, *coa* and *fnbA* genes are activated and the *cap* gene is repressed by the *sae* operon (Dassy et al., 1993; Wolz et al., 1996; Saravia-Otten et al., 1997; Luong et al., 2002; Steinhuber et al., 2003). Toxic shock syndrome toxin (TSST) is positively regulated by both *agr* and *sae* operon. However, *agr* mutants did not show impairment of TSST toxin production as observed in *sae* mutants (Baroja et al., 2016). Despite the positive regulation of *hla* expression *via* RNAIII activation by *agr* locus *in vitro, sae* operon has been reported to be inevitable for *in vivo* toxin production (Novick et al., 1993; Goerke et al., 2001).

(ii) **σ**_**B**_

In the case of *sae* operon, σ_B_ has been reported to cause downregulation of *saePQRS* and *sae* target genes (*hla, nuc, splABCDEF*, and *hlgABC*; Mitchell et al., 2013) mostly *via* regulatory proteins or small non-coding RNAs which may be positively regulated by σ_B_ (Bischoff et al., 2004). No evidence of cross-talk between *sae* locus and σ_B_ has been reported, suggesting the fact that both are independent regulatory systems (Goerke et al., 2005; Liu et al., 2016).

(iii) **CodY**

Branched-chain amino acids (BCAA) *viz*., isoleucine, leucine, valine, and GTP are the key metabolites that activate CodY as a DNA-binding protein and bind to the sequence motif (AATTTTCWGAAAATT) of chromosomal DNA. CodY and SaeR can both bind to the *sae* P1 promoter in an opposite manner where one is the repressor and the other is the activator, respectively. CodY and SaeR have been reported to compete for binding to the *saeP* regulatory region. CodY is a stronger repressor of *sae* P1 promoter as compared to Rot. When nutrients are abundant, CodY inhibits the transcription of *saeRS* operon and *sae* dependent virulence genes but with the depletion of nutrients, the affinity of CodY to *sae* P1 promoter is lost resulting in an upregulation of *sae* operon. The subsequent increase in the production of cytotoxic factors helps *S. aureus* to combat the host immune system by destroying neutrophils. Pendleton et al. (2022) have reported that the cell membranes of *codY* mutant strains of *S. aureus* have a higher percentage of branched-chain fatty acids (BCFAs) as compared to the cell membranes of wild-type strains. This observation suggests the possibility of post-transcriptional regulation of the Sae system by the global repressor codY. Disruption of the *lpdA* gene which encodes dihydrolipoyl dehydrogenase (an important enzyme in BCFA synthesis) results in a reduction of SaeS kinase activity as well as the response regulator SaeR-P. This ultimately leads to a reduction in exotoxin secretion and attenuation of virulence. Thus, CodY acts as a nutritional checkpoint protein which ensures that the *saePQRS* operon is activated only when cytolytic and pore-forming toxins are to be secreted to acquire nutrition from the host and functions as a switch between commensal and invasive lifestyles of *S. aureus* (Mlynek et al., 2018).

(iv) **WalRK**

WalRK is one of the major two-component system of *S. aureus* which plays a role in cell wall metabolism and cell viability (Dubrac and Msadek, 2004; Dubrac et al., 2007). When the response regulator, WalR is produced in large amounts in its active form then the upregulation of *sae* operon has been observed. However, this upregulation was terminated when SaeRS two component system was deleted (Delauné et al., 2012). These results indicated that WalRK positively affects SaeRS TCS; however, the exact mechanism by which it occurs is still unknown (Liu et al., 2016).

(v) **Fur regulon**

Fur regulon of *S. aureus* is responsible for iron uptake, biofilm formation, and anti-oxidative stress response (Hantke, 1981; Litwin and Calderwood, 1993; Horsburgh et al., 2001; Johnson et al., 2005; Richardson et al., 2006; Lee and Helmann, 2007). *saeRS* transcription from both P1 and P3 promoters is downregulated in *fur* mutant as well as when iron from an exogenous source is supplied in media (*fur* is downregulated as iron is freely available), suggesting that Fur may be a positive regulator of *saeRS* (Johnson et al., 2011). However, the direct effect of Fur regulon on *saeRS* transcription is still unclear because *sae* operon does not possess Fur binding sites (Cho et al., 2015; Liu et al., 2016).

(vi) **Rot**

Rot, a member of the SarA protein family, is known to repress the expression of toxins (such as *hla*) in *S. aureus* (McNamara et al., 2000; Li and Cheung, 2008). Li and Cheung (2008) have reported that Rot can bind to the P1 promoter of the *sae* locus and repress it suggesting that Rot represses *hla* transcription *via* the P1 promoter of the *sae* locus. However, as the P1 promoter is not involved in the transcription of *sae* target genes (Jeong et al., 2011), the observations of Li and Cheung (2008) seem to be unlikely (Liu et al., 2016).

(vii) **Fak system**

Fatty acid kinase (*fakA* and *fakB1/fakB2*) mutants of *S. aureus* have been reported to exhibit a decrease in α hemolysin production as well as other *sae* target genes, indicating positive regulation of SaeRS TCS by the Fak system. It is believed that the acyl-PO_4_ group of FakB may donate the phosphoryl group to SaeR leading to its activation (Parsons et al., 2014).

(viii) **ScrA**

A novel protein ScrA acts *via* the SaeRS TCS to regulate virulence gene expression in *S. aureus*. ScrA protein acts as an intermediate between ArlRS and SaeRS systems (Wittekind et al., 2022). ArlRS TCS regulates genes involved in adhesion (*ebh* and *sdrD*), virulence (*nuc, lukA*, and *esxA*), and transcriptional factors (*sarV* and *mgrA*; Crosby et al., 2020). *scrA* expression is increased directly *via* the ArlR response regulator or indirectly *via* other regulators such as *mgrA*. ScrA has been hypothesized to stimulate SaeS kinase activity. This activates the SaeRS TCS which upregulates the production of adhesins and hemolysins and downregulates proteases which in turn increases cellular aggregation, biofilm formation, and hemolysis. The activated SaeRS TCS acts as a feedback inhibitor and directly or indirectly represses *scrA* (Wittekind et al., 2022).

## 5. Conclusion

*S. aureus* being a normal microflora of humans will always coexist with mankind. The extensive use of antibiotics across the world has led to the emergence of more resistant strains such as MRSA requiring novel antibiotics and treatment strategies. The ease of infection, high mortality rates, lack of suitable animal models, and increase in antibiotic resistance in MRSA have proved to be major hurdles to advances in clinical research. *S. aureus* pathogenesis is more complex as it is not dependent on a single major virulence factor that leads to disease progression. Secretion of a diverse array of virulence factors during its course of infection poses a major challenge in both drug and vaccine development. Though humans have made outstanding achievements in understanding the pathogenesis of MRSA, there are still gaps in knowledge and some important challenges to overcome. First, the adaptive immune evasion mechanisms post *S. aureus* infections remain unknown. Second, a vaccine targeting multiple factors against *S. aureus* is yet not successfully developed. Apart from these, a better understanding of bacterial pathogenesis, prevention of transmission and infection, and advancement in diagnosis requires focused concentration by researchers, policymakers, funding agencies, and well-coordinated multidisciplinary approaches that may help control the transmission of this highly successful pathogen.

## Author contributions

HP has written the article. SR has designed the concept and edited the article. All authors contributed to the article and approved the submitted version.
